# Identification of lamprey variable lymphocyte receptors that target the brain vasculature

**DOI:** 10.1038/s41598-022-09962-8

**Published:** 2022-04-11

**Authors:** Jason M. Lajoie, Moriah E. Katt, Elizabeth A. Waters, Brantley R. Herrin, Eric V. Shusta

**Affiliations:** 1 Department of Chemical and Biological Engineering, University of Wisconsin-Madison, Madison, WI 53706 USA; 2 Department of Neurological Surgery, University of Wisconsin-Madison, 1415 Engineering Drive, Madison, WI 53706 USA; 3 Department of Pathology and Laboratory Medicine, Emory University, 1462 Clifton Rd NE, Atlanta, GA 30322 USA

**Keywords:** Biotechnology, Neuroscience, Neurology, Engineering

## Abstract

The blood–brain barrier (BBB) represents a significant bottleneck for the delivery of therapeutics to the central nervous system. In recent years, the promise of coopting BBB receptor-mediated transport systems for brain drug delivery has increased in large part due to the discovery and engineering of BBB-targeting antibodies. Here we describe an innovative screening platform for identification of new BBB targeting molecules from a class of lamprey antigen recognition proteins known as variable lymphocyte receptors (VLRs). Lamprey were immunized with murine brain microvessel plasma membranes, and the resultant repertoire cloned into the yeast surface display system. The library was screened via a unique workflow that identified 16 VLR clones that target extracellular epitopes of in vivo-relevant BBB membrane proteins. Of these, three lead VLR candidates, VLR-Fc-11, VLR-Fc-30, and VLR-Fc-46 selectively target the brain vasculature and traffic within brain microvascular endothelial cells after intravenous administration in mice, with VLR-Fc-30 being confirmed as trafficking into the brain parenchyma. Epitope characterization indicates that the VLRs, in part, recognize sialylated glycostructures. These promising new targeting molecules have the potential for brain targeting and drug delivery with improved brain vascular specificity.

## Introduction

The brain vasculature, also known as the blood–brain barrier (BBB), is substantially more impermeable to blood-borne constituents than the peripheral vasculature. Continuous paracellular tight junctions between brain endothelial cells (BECs) combine with a low level of pinocytosis to restrict the nonspecific brain uptake of molecules, proteins and cells, while a host of drug efflux transporters serve to actively pump molecules that do enter the BECs back into the bloodstream^1^. While these barrier functions are essential in health, they present a significant challenge when attempting to treat neurological disorders because the majority of small molecule therapeutics, and essentially all gene and protein-based drugs, do not appreciably cross the BBB^2^. Therefore, effective non-invasive drug delivery strategies that can overcome this barrier are critical for the successful development of central nervous system (CNS) therapeutics.

As a result of the prominent barrier function, the BBB endothelia express numerous transport systems to facilitate brain uptake of key nutrients such as glucose and amino acids as well as proteins such as transferrin and insulin^3^. Importantly, it is possible to coopt certain endogenous receptor-mediated transport systems, such as the transferrin receptor (TfR), and insulin receptor (IR), for the delivery of drug payloads across the BBB using receptor-targeting antibodies or ligand mimics^4^. Although pharmacologic amounts of drug can be successfully delivered to the brain by targeting these receptors, several factors combine to limit their efficiency. Ubiquitous expression of TfR and IR throughout the body results in mis-targeting to peripheral organs, limiting brain uptake and increasing the potential of peripheral effects^5,6^. Furthermore, affinity- and avidity-based interactions can result in lysosomal degradation of antibodies within the BECs, further limiting access to the brain^7,8^. While efforts to engineer the binding properties of TfR targeting antibodies has shown some success^9–12^, typically less than 1% of the injected dose of therapeutic antibody reaches the brain parenchyma requiring high concentration dosing (up to 50 mg/kg). Thus, there remains a significant need for discovery and development of novel BBB receptor-targeting antibodies.

Genomic and proteomic profiling of BECs is one approach that has been implemented to identify new BBB transport systems as described fairly recently where highly expressed proteins basigin and CD98 heavy chain were identified as potential drug carriers^13^. However, it is often difficult to determine what BBB receptors are actually capable of transport simply from sequence data as non-canonical transporters have been identified^13–15^. An alternative approach involves phenotypic screening of large combinatorial antibody or peptide repertoires both in vitro and in vivo to identify BBB targeting molecules^16^. However, despite considerable screening efforts, few new targeting reagents have been generated^16^. For example, there has been limited success using in vivo screening approaches, such as the systemic injection of phage libraries^17^, likely due to high phage background binding, immune clearance, and significant phage uptake by peripheral organs. Furthermore, promising clones identified from in vitro screening platforms, such as phage and yeast display biopanning on cultured cells, often do not cross-react with in vivo antigens^18^ as a result of altered expression profiles of BECs in the petri dish^19,20^. To date, the antibody repertoires employed have been limited to nonimmune mammalian antibody fragments, while application of immunization-based approaches in the BBB field have been confined to targets arising from genomic proteomic profiling efforts^14,21–25^. Therefore, the search for new BBB targets would benefit from the development and application of innovative screening platforms with the ability to more broadly and efficiently sample the in vivo-relevant BBB antigen landscape.

In this work, we describe a new screening workflow to address many of the aforementioned challenges. We deployed a family of highly diverse leucine-rich repeat proteins termed Variable Lymphocyte Receptors (VLRs) that function as antigen receptors in the adaptive immune system of lamprey^26–28^. VLRs possess diversity, specificity, affinity, and stability comparable to traditional Ig-based antibodies^29,30^. Lampreys last shared a common ancestor with mammals greater than 500 million years ago. This tremendous phylogenetic distance, combined with the unique crescent-shaped geometry of the antigen-binding site, may enable VLRs to recognize new antigenic targets, including highly conserved proteins and carbohydrates that may not be well recognized by mammalian antibodies^31–36^. For example, the BBB glycocalyx has been shown to play significant roles at the BBB in health and disease^25,37–39^, and yet has largely not been exploited as an antigenic source for BBB targeting. In an attempt to leverage these potential advantages for BBB targeting, lampreys were immunized with BEC plasma membranes fractionated from mouse brain microvessels to create a library of VLRs against in vivo-relevant BBB antigens, and the immune VLR repertoire was subsequently imported into the yeast surface display (YSD) platform for screening. The immune VLR library was first screened against detergent-solubilized versions of the same brain microvessel plasma membrane preparations used for immunization to ensure enrichment of clones against in vivo antigens. Subsequently, biopanning of this enriched pool on cultured mouse BECs was used to enrich VLRs targeting extracellular epitopes. In vitro cell-binding and internalization assays revealed a subset of VLRs capable of internalization and trafficking within BECs. Three lead VLRs with prominent glycan-binding signatures were identified and shown to target and traffic within and across BECs in vivo.

## Results

### Lamprey immunization and yeast display library construction

**Figure 1:**
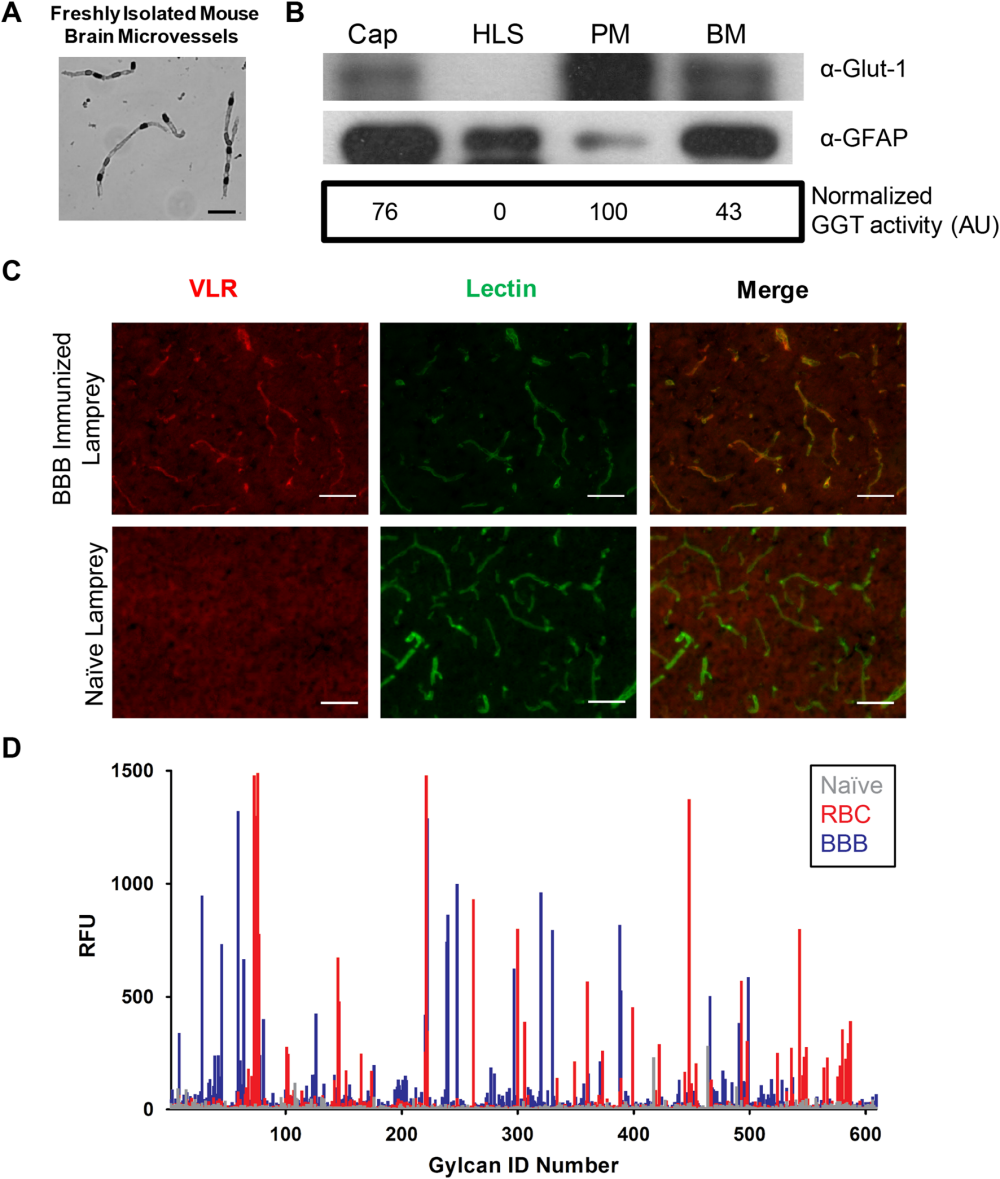
Brain microvessel plasma membrane (BMPM) isolation and lamprey immunization validation. (**A**) Isolated mouse brain microvessels were stained with trypan blue. Scale bar = 25 μm. (**B**) Cropped western blots for Glut-1 and GFAP along with normalized GGT activity for the various fractions generated during BMPM isolation. Raw western blot data can be found in Fig. S11. Hypotonic lysis was used to disrupt the isolated brain microvessels. Following centrifugation, the supernatant (HLS) was separated from the lysed microvessel fragments. Sonication of the microvessel fragments and centrifugation separated the plasma membranes (PM) from the basement membrane pellet (BM). (**C**) Pooled plasma from BBB immunized lampreys or a plasma sample from a naïve lamprey were used to immunolabel mouse brain sections (red) and brains were counterstained with fluorescent IB_4_-lectin (green) to identify brain microvessels. Scale bar = 50 μm. (**D**) Glycan microarray analysis. Plasma samples from a naïve lamprey (Naïve, gray), lamprey immunized with human erythrocytes (RBC, blue), or BMPM immunized lamprey (BBB, red) were used to probe the CFGv5.2 glycan microarray. RFU = relative fluorescence units.

Brain microvessels were isolated from mice cortices using mechanical homogenization and filtration techniques (Fig. 1A). Plasma membranes (PM) were fractionated from the basement membranes of the microvessels via hypotonic lysis, sonication and centrifugation as previously described^39,40^. Assessment of the preparations indicated that the endothelial PM-resident glucose transporter (Glut-1) and gamma-glutamyl transpeptidase (GGT) enzyme were enriched in the PM fraction while the astrocyte marker, glial fibrillary acidic protein (GFAP), was de-enriched (Fig. 1B). Such a brain microvessel PM (BMPM) antigen preparation has been previously used as an appropriate immunogen for multiplex expression cloning of BBB membrane proteins^26^, and was used here as a representative in vivo-relevant BBB source for lamprey immunization and VLR library screening.

**Figure 2:**
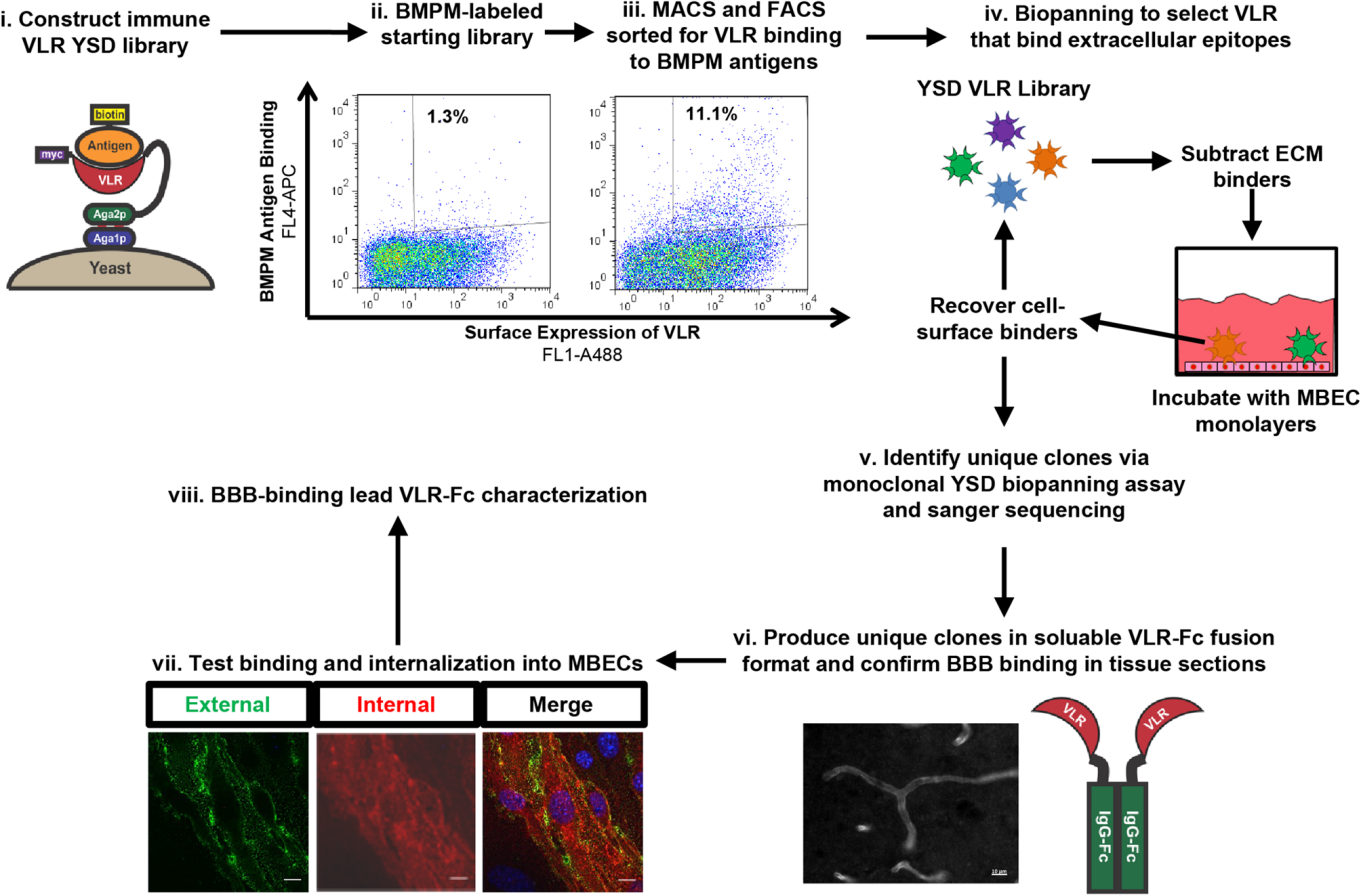
BBBVLR library screening and characterization workflow. (i) Immunized lamprey BBBVLR libraries are expressed on the yeast surface by fusion to the C-terminus of the Aga2p protein. Each yeast cell displays thousands of copies of a single VLR clone on its surface. (ii) and (iii) The immune, unsorted library is enriched for binders to BBB antigens expressed in vivo via MACS and FACS with detergent-solubilized BMPMs. The percentage of antigen-binding yeast clones before (ii) and after (iii) sorting are reported. (iv) The FACS-enriched BMPM-binding pool is next screened for VLRs that bind extracellular domains of BMPM epitopes via biopanning against an MBEC cell line while also employing negative selection against ECM in each round. (v) Unique MBEC binding clones are identified via a monoclonal biopanning and sequencing workflow. (vi) Unique VLRs are reformatted as rabbit IgG-Fc fusion proteins, secreted from HEK293 cells, and used to immunolabel brain sections to verify binding to relevant BBB antigens in the mouse brain. (vii) VLR capability for BBB transport is assessed in vitro with an internalization assay using cultured MBECs. (viii) Lead VLR-Fcs coming out of this pipeline are further characterized in various in vitro and in vivo assays.

Three lampreys were immunized with 50 μg of BMPM protein and boosted two additional times over 6 weeks to elicit a VLR immune response against mouse BBB antigens. Two weeks after the final immunization, lamprey plasma and lymphocytes were harvested. B-cell-like lymphocytes that produce soluble VLR type B (VLRB) proteins in response to adaptive challenge were the focus of this study^41^. Mouse brain cryosections were labeled using pooled plasma from the BBB immunized lampreys to determine if the lampreys responded to the immunization. Brain microvessels were clearly labeled with the polyclonal VLRB-containing plasma pooled from all three immunized lampreys (Fig. 1C), whereas plasma from naïve lamprey did not elicit a vascular-specific signature. These results indicated that the lamprey immune system generated a specific response against BMPM antigens. In addition, when the VLRB-containing plasma was used to probe a glycan microarray, there was a distinct glyco-signature for the BMPM immunized lampreys compared to human red blood cell immunized lampreys or naïve lampreys (Fig. 1D). To efficiently screen for monoclonal BBB-binding VLRs present in the immune repertoire, a YSD library termed BBBVLR, was constructed (Fig. 2i). VLR genes were recovered by PCR of total lamprey lymphocyte cDNA with VLRB-specific primers and assembled by homologous recombination with the YSD vector to create an immune VLR YSD library of 7.5 × 10^6^ VLR clones (See “Materials and methods” for details).

### Screening of VLR library

**Figure 3:**
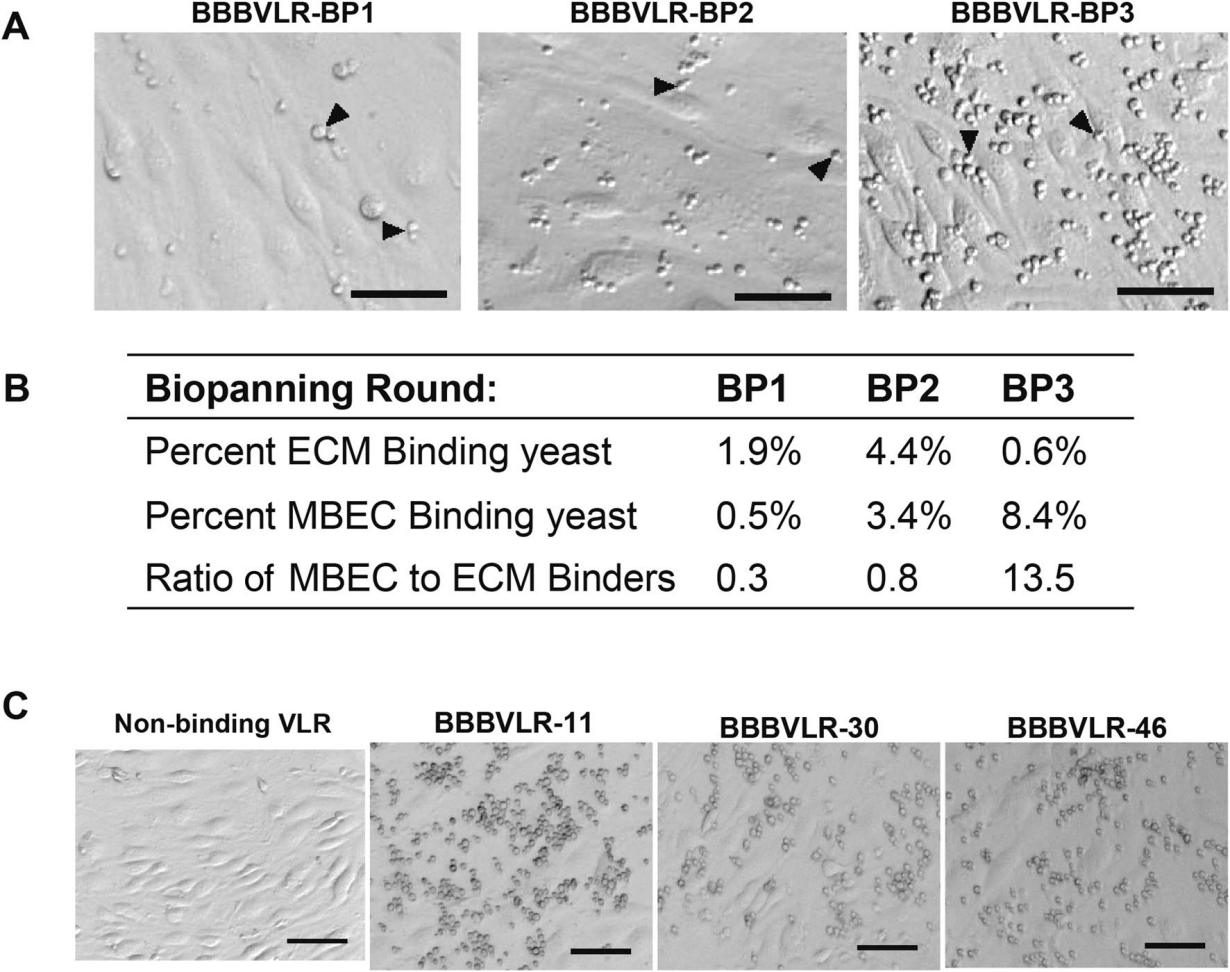
YSD screening of the BBBVLR library. (**A**) Representative brightfield images of yeast binding to confluent MBEC monolayers at the end of each biopanning round. Yeast are the small round cells and examples are indicated by black arrowheads. (**B**) Percentage of applied yeast that were recovered by binding to ECM and MBEC during each round of biopanning. (**C**) After the third round of biopanning, MBEC-binding VLR clones were identified via a 96-well biopanning assay using yeast displaying RBC36 VLR as a non-binding VLR control. Scale bars = 50 μm.

A two-tiered screening strategy was designed and implemented to isolate VLRs from the BBBVLR library that bind to in vivo BBB cell-surface antigens (Fig. 2). The library was initially screened for binding to in vivo antigens via a modified yeast display immunoprecipitation (YDIP) method^40,42^ using biotinylated, detergent-solubilized mouse BMPM proteins as the source of antigens for library sorting (Fig. 2ii-iii). Subsequently, YSD biopanning^18^ on an immortalized mouse brain endothelial cell (MBEC) line, bEnd.3^43^, was carried out to recover VLRs that bind to extracellular epitopes as required for BBB targeting (Fig. 2iv). In more detail, two rounds of YDIP screening with freshly isolated BMPM antigen preparations were carried out, with one round of magnetic activated cell sorting (MACS) followed by one round of fluorescence activated cell sorting (FACS). The resultant library had an approximately 10-fold enrichment in the percentage of BMPM antigen-binding yeast over the starting library (Fig. 2ii-iii). Next, three rounds of YSD biopanning were carried out (Figs. 2iv and 3A,B). Given the nature of the BMPM preparations, there existed VLR clones in the FACS-enriched pool that bound to BBB extracellular matrix (ECM) (Fig. S1). Therefore, each biopanning round included a subtraction panning step to remove ECM binders from the library. Yeast pools were first incubated on decellularized ECM derived from confluent MBEC cultures to subtract ECM binders from the library^44^. Subsequently, non-ECM binding yeast were recovered and immediately applied to intact MBEC monolayers. After washing steps, clones that remained bound to the MBEC cell surface were recovered and regrown for subsequent rounds of biopanning or monoclonal analysis. The biopanning approach enriched for MBEC cell surface binding yeast and de-enriched for ECM binders over three rounds as shown in Fig. 3A,B. Initially, there was a significant over-representation of ECM binders compared to MBEC binders as shown by the 0.3 MBEC:ECM binder ratio in the BBBVLR-BP1 pool (Fig. 3B). By the final round of biopanning a very small percentage of the library bound to the ECM plate (0.6%, Fig. 3B) relative to the MBEC binding population (8.4%) representing a nearly 50-fold enrichment in MBEC specific binders over ECM binders. Subsequently, individual clones were tested in a 96-well biopanning assay and 204 out of 240 clones were found to specifically interact with MBEC cells by light microscopy as shown for a select group of clones in Fig. 3C. Sequencing of the VLR genes revealed that 33 of the 204 cell-binding clones represented unique VLR amino-acid sequences.

### VLR clones bind to in vivo-relevant BBB antigens

**Figure 4:**
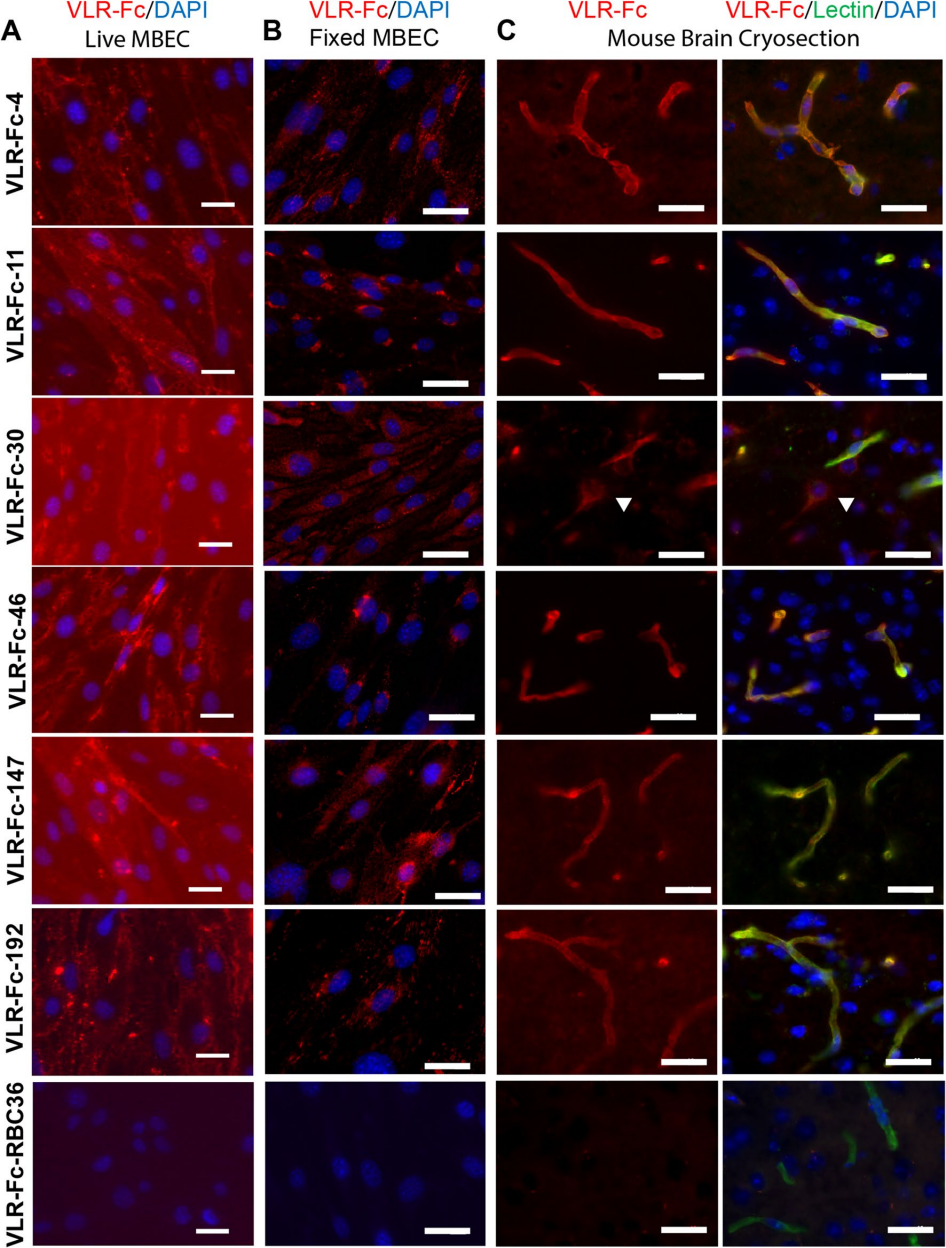
BBB binding profiles for individual VLR clones. VLRs were produced as VLR-Fc fusions and used for immunolabeling (red) of (**A**) Live MBECs, (**B**) Fixed and permeabilized MBECs, and (**C**) Mouse brain cryosections. Labeling with IB_4_-lectin is used to denote the location of microvessels (green). VLR-Fc-RBC36 was used as an isotype control. A white arrowhead highlights binding of VLR-Fc-30 to parenchymal cells. In panels A-C, DAPI (blue) is employed as a nuclear counterstain. Scale bars = 25 μm.

To validate that the screening strategy described above yielded MBEC-targeting VLRs that also bound to antigens expressed at the in vivo BBB, the 33 unique VLR clones were analyzed for MBEC and BBB binding. VLRs were produced in transiently transfected HEK293 cells as dimeric, recombinant Fc fusion proteins (VLR-Fc) (Figs. 2vi and S2). Of the 33 clones, 26 bound to the surface of live MBEC cells (Fig. 4A and Table S1). The remaining 7 clones were either produced at insufficient levels or binding was not detected. Fixed and permeabilized MBEC cells were also labeled with VLR-Fc clones to reveal subcellular localization patterns, of which several VLRs bound differentially to prominent intracellular antigen pools (Fig. 4B), suggestive of internalizing receptors. Next, mouse brain cryosections were labeled with VLR-Fcs to evaluate binding to antigens expressed in vivo (Fig. 4C). Notably, the majority of the MBEC binding VLR clones (19 of 26) were shown to bind antigens in mouse brain. In terms of brain localization, 14 of 19 VLR-Fcs appeared to be selective for the brain vasculature (Table S1 and Fig. 4C, e.g. VLR-Fc-4, 11, 46, 147, and 192), whereas, 2 bound both vascular and parenchymal antigens (Table S1 and Fig. 4C, e.g. VLR-Fc-30 white arrowhead). Further evaluation of the parenchymal cell target of VLR-Fc-30 indicated that many of the postvascular cells recognized by VLR-Fc-30 are NeuN positive neurons (Fig. S4). In addition, 3 VLR bound only parenchymal antigens (Table S1, e.g. VLR-Fc-5). Finally, select lead candidates, VLR-Fc-11, -30, and -46, also bound the human brain vasculature with VLR-Fc-11 and -46 binding selectively to blood vessels and VLR-Fc-30 binding to blood vessels and parenchymal cells (Fig. S5). In summary, a rather large set of 16 VLRs displaying diverse MBEC surface and intracellular binding patterns as well as brain section binding were identified using this screening strategy.

### A subset of VLRs are internalized by MBECs in vitro

**Figure 5:**
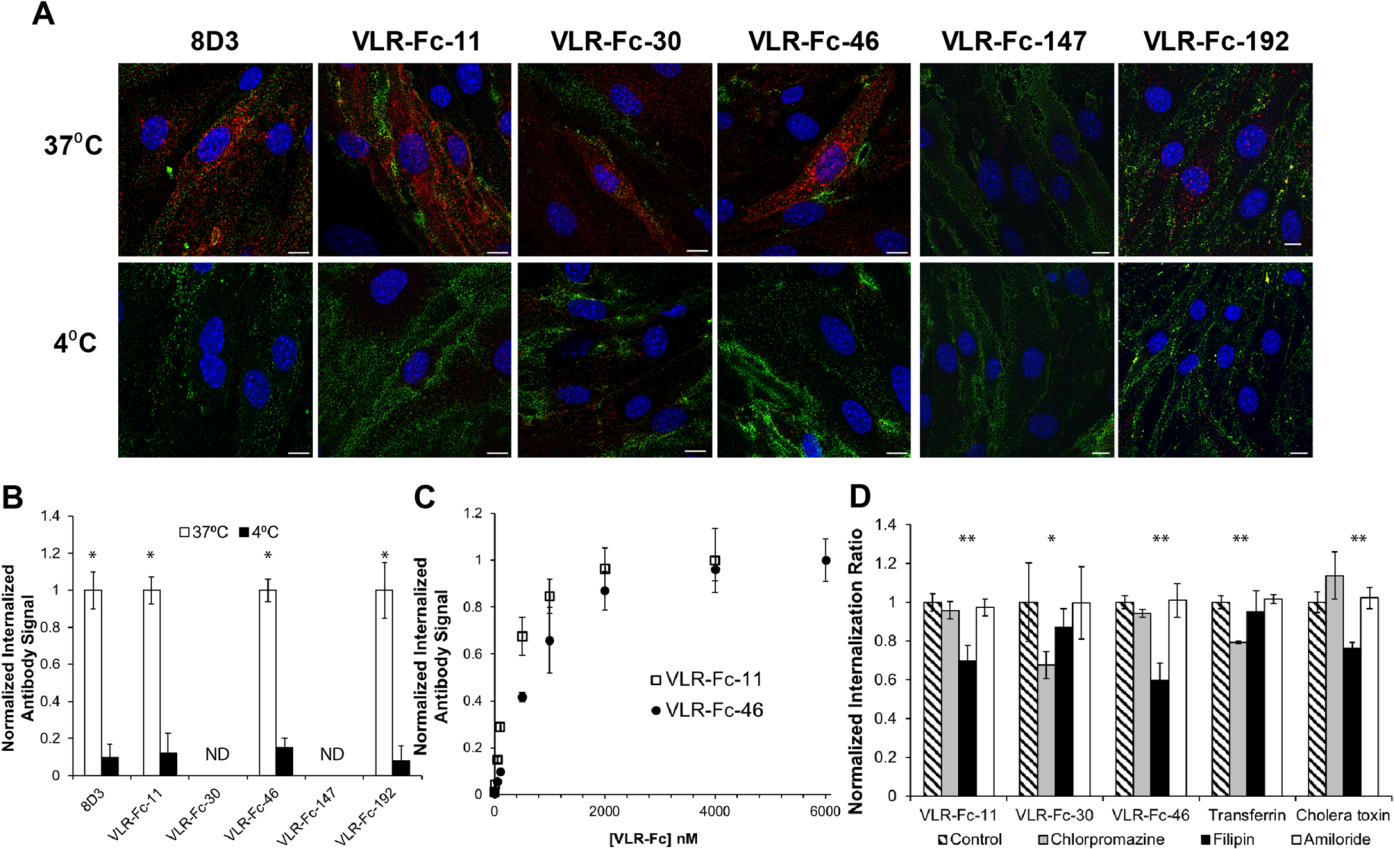
MBEC internalization of VLR-Fcs. (**A**) MBEC monolayers were incubated with either anti-TfR antibody (8D3) or the indicated VLR-Fc for 30 min at 37 °C or 4 °C. Surface bound (green) and internalized (red) antibody pools were differentially labeled. Representative confocal Z-slices are shown. DAPI (blue) was employed as a nuclear counterstain. Scale bars = 10 μm. (**B**) Per cell VLR-Fc internalization at 37 °C and 4 °C was quantified using a LiCor scanner and normalized to the 37 °C signal for each clone. ND = Not Detected. (**C**) Saturability of the internalization pathway was evaluated by titrating VLR-Fc concentrations and quantifying MBEC internalization as in (**B**). (**D**) Normalized ratio of internalized to surface bound VLR-Fcs in the presence of inhibitors of endocytosis pathways. For each sample, ratios were normalized to that of the no treatment control using immunocytochemistry quantification approaches (see “Materials and methods” for details). Transferrin and cholera toxin, were employed as assay positive controls for chlorpromazine and filipin inhibition, respectively. Data in B, C, and D represent the mean ± S.D. of 3 independent internalization or saturation experiments (**B**). A two-tailed students *t* test on n = 3 replicates was used to determine statistical significance. (**D**) One-way ANOVA paired with Tukey’s post-hoc analysis on n = 3 replicates was used to determine statistical significance. *p < 0.05, **p < 0.01.

Next, in vitro internalization assays were used to further characterize the 16 VLR-Fc clones described above for properties compatible with brain drug delivery applications (Fig. 2vii). Initially, the VLR-Fc were subjected to a temperature-based internalization assay employing a two-color labeling procedure to differentiate cell surface versus internalized VLR-Fc (See “Materials and methods” for details). Endocytosis requires membrane fluidity and is inhibited at 4 °C; thus, comparison of internalization of VLR-Fc at 4 or 37 °C provided evidence for a membrane-dependent endocytosis process. Using this approach, 4 out of 16 VLR-Fc tested were shown to internalize via a temperature-dependent process (Fig. 5A,B). VLR-Fc-11, VLR-Fc-30, VLR-Fc-46, and VLR-Fc-192 were endocytosed at 37 °C evidenced by intracellular VLR-Fc signal; whereas, VLR-Fc-147 only bound to the cell surface (Fig. 5A). Furthermore, internalization was inhibited at 4 °C for the 4 VLR-Fcs as only surface-bound antibodies were detected (Fig. 5A). In all cases, the internalized VLR-Fc signal was observed in punctate structures within the cytoplasm, indicative of endocytic vesicle trafficking and similar to punctate structures observed for a control anti-transferrin receptor antibody, 8D3, known to internalize into BECs. While VLR-Fcs-11, -46 and -192 were internalized into nearly all the cells on the plate, VLR-Fc-30 only internalized into a small subset of cells despite exhibiting a cell surface binding signal throughout the plate (Fig. S3). Antibody internalization was also quantified via labeling and detection of intracellular antibody after an acid wash to remove surface bound proteins, and the results agreed with the confocal analysis with roughly 10-fold higher amounts of internalized antibody at 37 °C (Fig. 5B). As mentioned above, since VLR-Fc-30 internalizes in only a subset of cells, it was not detectible in this aggregate assay. Another hallmark of receptor-mediated endocytosis is saturability. Given a finite number of cellular receptors and associated trafficking machinery, the amount of ligand internalized will saturate with increasing ligand concentration. Indeed, saturation of the internalization pathway was observed for VLR-Fc-11 and VLR-Fc-46 at antibody concentrations around 2 µM (Fig. 5C), values similar to that for saturation of the transferrin receptor system^44^. In addition to saturability, VLR-Fc-11, -30, and -46 were incubated with MBECs in the presence of endocytosis inhibitors to determine the predominant pathway of endocytosis (filipin-caveolin, chlorpromazine-clathrin, amiloride-macropinocytosis). VLR-Fc-11 and -46 showed significant decreases in internalization with filipin, indicating that caveolin-mediated endocytosis contributed to the trafficking of VLR-Fc-11 and -46. By contrast, VLR-Fc-30 exhibited decreased internalization with chlorpromazine suggesting VLR-Fc-30 internalization by clathrin-dependent mechanisms (Fig. 5D). Based on their ability to bind to and traffic within MBECs via endocytosis, VLR-Fc-11, -30, and -46 were selected as lead candidates for additional in vitro antigen-binding characterization and in vivo analysis. As described later, the in vivo analysis indicated that VLR-Fc-192 did not target the brain vasculature after systemic administration so it was not characterized further.

### Antigen-binding characterization reveals a role for glyco-recognition in cell binding

**Figure 6:**
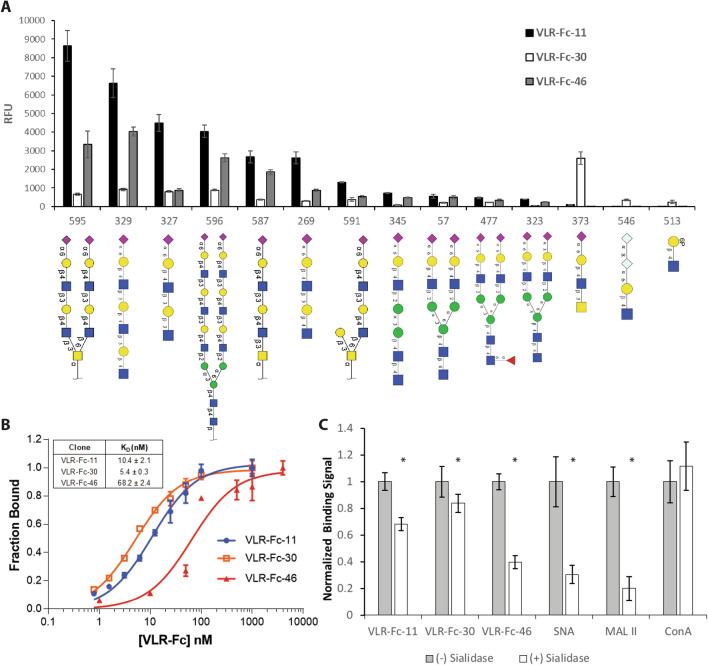
Antigen-binding characterization of lead VLR-Fc clones. (**A**) VLR-Fc binding to glycans on the CFGv5.3 mammalian glycan array. Comparison of binding strength of the top 10 glycans recognized by each VLR-Fc is shown and rank ordered by VLR-Fc-11 binding intensity. The glycan ID number and structure are shown along the x-axis. RFU = relative fluorescence units is plotted as mean ± S.D. Raw data used to generate this plot can be found in Table S2. (**B**) Apparent affinity of each VLR-Fc was measured via equilibrium titration on cultured MBECs. The mean fitted equilibrium dissociation constant (K_D_) ± the 95% confidence interval calculated from n = 3 independent titrations is reported. (**C**) VLR-Fc or lectin binding to MBEC monolayers with and without sialidase pre-treatment. For each protein tested, the background-subtracted binding signal is normalized to MBEC binding without sialidase pre-treatment. VLR-Fc are compared to lectins that bind α2-3 (MAL II) or α2-6 (SNA) linked sialic acids and ConA which binds to α-linked core mannose residues. Bars with the mean ± S.D. are plotted. A two-tailed students t-test on n = 6 replicates was used to determine statistical significance *p < 0.05. SNA = *Sambucus nigra* lectin, MAL II = *Maackia amurensis* lectin 2, ConA = Concanavalin A.

Given that VLRs have proven robust in their capability to specifically bind glycan structures^31,32,45,46^, the potential role of glyco-recognition in the binding of VLR-Fc-11, -30, and -46 was investigated. First, the glycan binding specificities for the VLR-Fcs were evaluated using the Consortium for Functional Glycomics (CFG) glycan microarray which comprises 600 validated mammalian glycans^46^, and there was a clear glycan binding signature for each of the VLRs. A comparison of binding profiles for the top 10 glycans recognized by each of the VLR-Fcs is shown along with their respective glycan structures (Fig. 6A and Table S2). VLR-Fcs -11 and -46 exhibited a similar rank ordering and a clear preference for terminal α2-6 linked sialic acid structures having the Neu5Acα2-6Galβ1-4GlcNAc motif with a preference for a β1-3Gal linkage after the GlcNac residue (as seen in Glycan ID 595, 329, and 327). On the other hand, the binding profile of VLR-Fc-30 appears to be distinct with a Neu5Acα2-6Galβ1-4GlcNAcβ1-3GalNAc motif (seen in Glycan ID 373) not recognized by VLR-Fc-11 or -46 yielding the highest binding signal. In addition, VLR-Fc-30 displayed weaker binding (5- to 10-fold decrease in signal intensity versus VLR-Fc-11, Table S2) to the Neu5Acα2-6Galβ1-4GlcNAcβ1-3Gal motif (seen in Glycan ID 595, 329, and 327) preferred by VLR-Fcs-11 and -46. The similarities in glyco-specificity were supported by an examination of the VLR sequences which indicated high homology between VLRs 11, 30 and 46 with the only differences located in the LRRNT and LRR1 VLR domains (Fig. S6). These differences in VLR amino acid sequence could also contribute to differences in antigen binding affinity. Thus, the apparent affinity of VLR-Fc-11, -30, and -46 was determined by direct titration of VLR-Fcs onto MBECs. All three clones bound their cell surface antigens with apparent affinities in the nanomolar range (Fig. 6B). Interestingly, VLR-Fc-11 exhibited an approximately 7-fold higher affinity compared to VLR-Fc-46 (K_D_ = 10 nM and 68 nM respectively) which correlated with the signal intensities observed in the glycan microarray analysis where VLR-Fc-11 binding was routinely 2- to 5-fold higher than VLR-Fc-46. Taken together, the glycan array and affinity data suggest that the amino acid differences between VLR-Fc-11 and -46 mainly contribute to differences in affinity. On the other hand, the differences in the VLR-Fc-30 sequence may contribute to altered or broadened specificity preferring a Neu5Acα2-6Galβ1-4GlcNAcβ1-3GalNAc motif over Neu5Acα2-6Galβ1-4GlcNAcβ1-3Gal motif.

To augment the glycan array results and demonstrate that glycan binding is involved in the observed interactions with MBECs, VLR-Fc binding to MBECs was quantified with and without pretreatment of the cells by α2-3,6,8 sialidase which cleaves terminal α2-3, α2-6, or α2-8 linked sialic acid residues from glycans (Fig. 6C). Binding of both VLR-Fc-11 and VLR-Fc-46 to MBECs was significantly decreased after treatment with sialidase. MBEC binding of VLR-Fc-30 upon sialidase treatment was also decreased but to a lesser extent. These results correlated well with the glycoarray results where VLR-Fc -11 and -46 indicated a strong preference for a terminal sialic acid motif whereas the VLR-Fc-30 was less dependent on this motif. Moreover, the decrease in binding for VLR-Fc-11 and -30 upon sialidase treatment was less than that observed for lectins with known sialic acid binding specificity, SNA and MAL II (Fig. 6C), suggesting additional glycan or proteinaceous components to the antigenic epitopes. To rule out VLR-Fc recognition of BBB receptors such as the transferrin receptor (rTfR), Insulin receptor (rIR), and low-density lipoprotein receptor (rLDLR) that have been extensively evaluated for their drug delivery capability, a competitive binding assay was employed. VLR-Fcs were pre-incubated with excess recombinant receptor ectodomains prior to a live MBEC cell surface binding assay. MBEC binding was not affected for any of the VLR-Fcs upon receptor competition (Fig. S7). In contrast and as expected, competition with the rTfR protein reduced the anti-transferrin receptor antibody (8D3) binding signal to ~ 20% of the non-competition signal, whereas rIR or rLDLR competition did not inhibit 8D3 binding. These results suggest that VLR-Fc-11, -30, and -46 do not bind TfR, IR, or LDLR.

### VLRs target and traffic the brain vasculature after systemic administration

**Figure 7:**
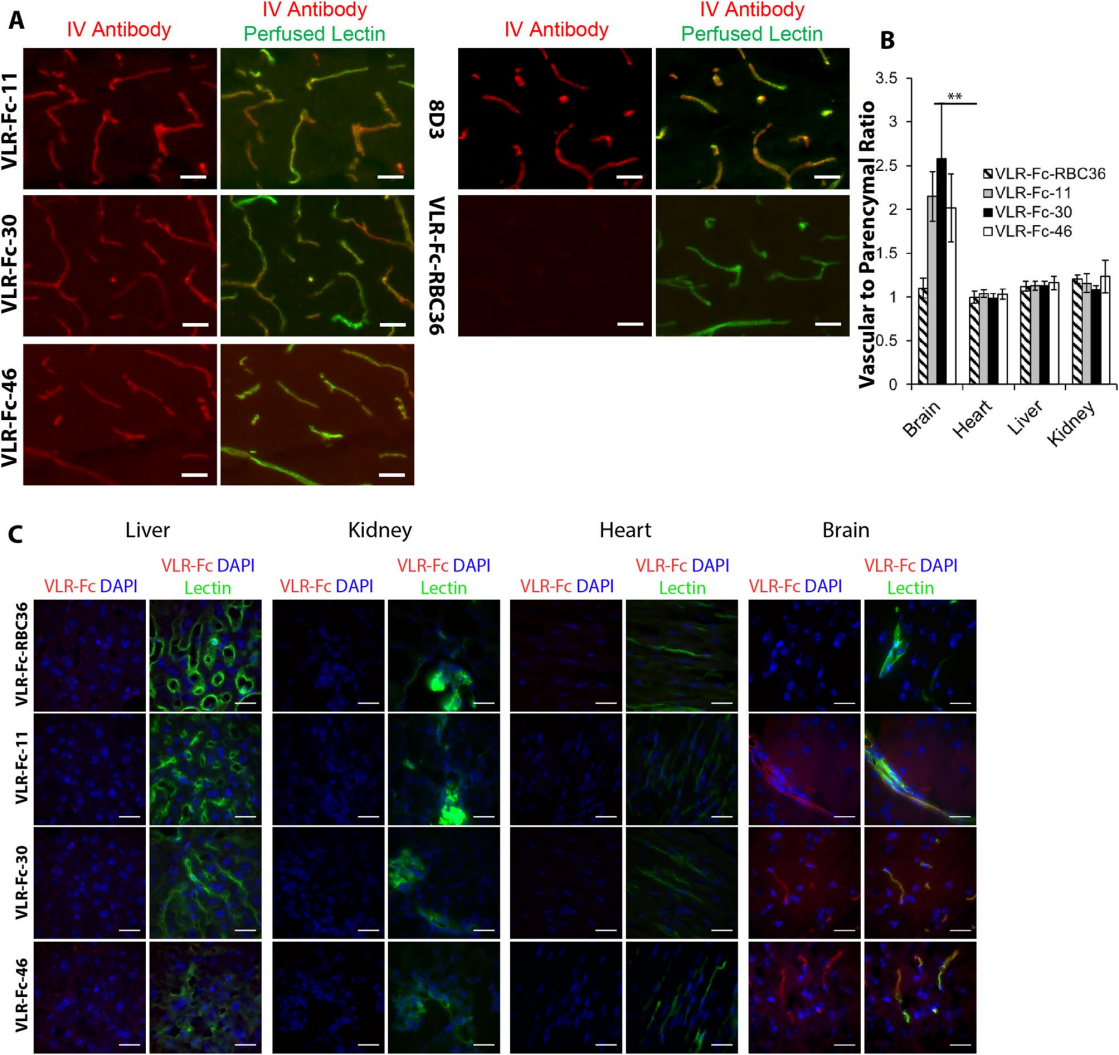
Vascular targeting and trafficking of VLR-Fcs after IV injection. Mice were administered 10 mg/kg of the indicated antibody construct (red). After 1 h of circulation time, mice were perfused with saline containing fluorescently labeled lectin (green) to remove unbound VLR-Fc and label the vascular lumen. (**A**) Low magnification fluorescence microscopy images from brain cortex. anti-TfR 8D3 antibody was used as a positive control for BBB targeting and VLR-Fc-RBC36 as an isotype control. Scale bars = 25 μm. (**B**) Ratio of vascular to parenchymal VLR signal, mean ± standard deviation, *p* < 0.01 = **determined with one-way ANOVA paired with Tukey’s post-hoc analysis. At least 5 different regions were imaged for each organ. (**C**) Representative images of vascular beds within the liver, kidney, heart, and brain. Scale bars = 25 μm.

We next sought to determine whether VLR-Fc could target the BBB after systemic administration. To this end, VLR-Fc-11, -30, -46, and -192 were administered intravenously (IV) to mice at a dose of 10 mg/kg and allowed to circulate for 1 h. The mice were then perfused through the left heart ventricle with saline containing fluorescently labeled lectin to both clear the vasculature of unbound antibody and stain the luminal aspect of microvessels for subsequent imaging analysis. Examination of brain cryosections from VLR-Fc injected animals clearly indicated that all VLR-Fc clones except VLR-Fc-192 (Fig. S8) bound to the brain vasculature and co-localized with the perfused vascular lectin stain, as did the 8D3 anti-TfR IgG control (Fig. 7A). In contrast, the isotype control VLR-Fc-RBC36 exhibited negligible residual background, despite strong vascular lectin labeling, indicating perfusion was effective in removing any unbound circulating VLR-Fc. Therefore, vascular-localized VLR-Fc signal observed for VLR-Fc-11, -30, and -46 was a result of these VLR-Fc engaging their target antigens on the brain vasculature. To get an idea of brain selectivity of VLR-Fc targeting, uptake in the vascular beds of other organs was also evaluated after VLR-Fc injection. There was no discernable vascular labeling above the VLR-Fc-RBC36 negative control for any of VLR-Fc-11, -30, or -46 in the heart, liver, or kidney, suggesting brain vascular targeting selectivity (Fig. 7B,C). Labeling of naïve, uninjected organ cryosections with VLR-Fc-11, -30, or -46 similarly showed no vascular binding above the VLR-Fc-RBC36 background (Fig. S9).

**Figure 8:**
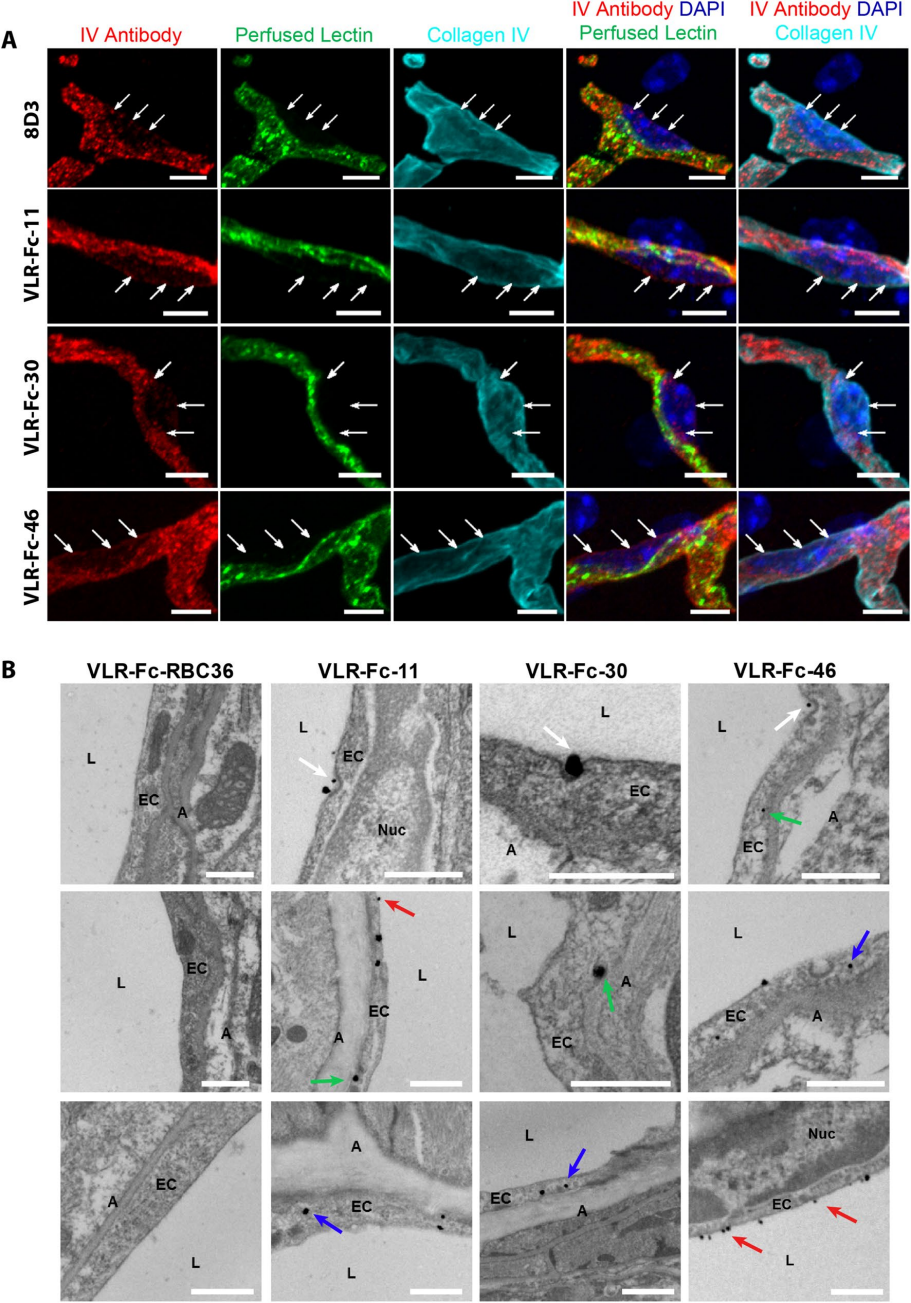
Vascular targeting following IV injection. Mice were administered 10 mg/kg of the indicated antibody construct. After 1 h of circulation time, mice were perfused with saline containing fluorescently labeled lectin to remove unbound VLR-Fc and label the vascular lumen. (**A**) Confocal microscopy analysis showing maximum intensity projections of ~ 7 μm Z-stacks. Post-labeling with anti-Collagen IV antibody (cyan) was used to delineate the microvessel basement membrane on the abluminal face of the vessels while DAPI (blue) was employed as a nuclear counterstain. VLR-Fc are observed in perinuclear punctate structures and along the abluminal side of the nucleus (white arrows). Representative images from the cortex are shown for n = 3 injected animals. Scale bars = 5 μm. (**B**) Immunogold electron microscopy localization of VLR-Fcs in MBECs after IV injection. Anti-rabbit Fc immunogold staining and silver enhancement reveals VLR-Fc localization (black electron dense spheres). VLR-Fc-11, -30, and -46 are observed binding at the luminal face of MBECs (red arrows) and within invaginating endocytic pits (white arrows). VLR-Fc are also observed within MBECs (blue arrows), and at the abluminal membrane (green arrows). VLR-Fc-RBC36 was not observed associated with MBECs. Scale bars = 500 nm. EC = brain endothelial cell, L = vessel lumen, Nuc = nucleus, A = abluminal side of brain ECs.

Next, we explored VLR-Fc trafficking at the BBB with higher magnification fluorescence and electron microscopic imaging. Confocal microscopy images indicate a punctate distribution of VLR-Fc co-localized with the luminal vascular lectin stain (Fig. 8A). Given the resolution limits of the confocal techniques used here (~ 100 nm per pixel) and that the MBECs are very thin (~ 100 nm), one can only separate the luminal and abluminal signals where the endothelial nuclei spread these two membranes further apart. In these regions, each of the VLR-Fc-11, -30 and -46 could be found in puncta at the abluminal membrane separated from the luminal lectin stain and co-localizing with the basement membrane (Fig. 8A). Similar results were observed with the 8D3, anti-TfR IgG control. These results were suggestive of VLR-Fc trafficking within the MBECs. To further confirm the MBEC trafficking capability of VLR-Fc-11, -30 and -46, immunogold electron microscopy was used to examine the ultrastructural localization of IV injected VLR-Fc at the brain microvasculature. VLR-Fc-11, -30 and -46 were all found localized to the luminal surface, in invaginating endocytic pits, within the cytoplasm of MBECs and at the abluminal membrane (Fig. 8B). By contrast, the isotype control VLR-Fc-RBC36 was not observed associated with the brain vasculature. Combined with the confocal microscopy analyses, these data indicate that VLR-Fc-11, -30 and -46 bind to the brain microvasculature and traffic across MBECs after systemic administration.

**Figure 9:**
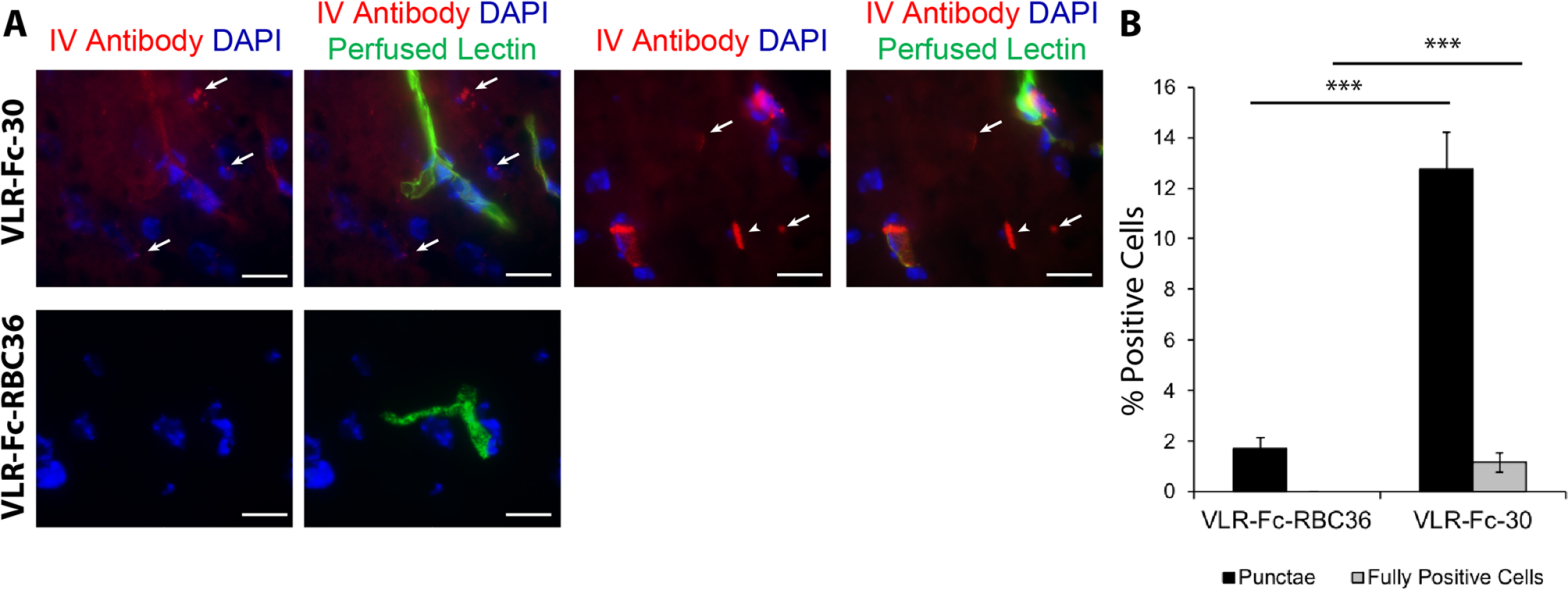
Mice were administered 10 mg/kg of the indicated antibody construct (red). After 11 h of circulation time, mice were perfused with saline containing fluorescently labeled lectin (green) to remove unbound VLR-Fc and label the vascular lumen. (**A**) Postvascular accumulation of VLR-Fc-30 was visualized as punctae (full arrows) and fully positive cells (arrow heads). Scale bars = 15 μm. (**B**) Quantification of percentage of DAPI positive cells exhibiting post vascular uptake of VLR-Fc-30 compared to VLR-Fc-RBC36 n = 3 injected animals, a total of 1007 and 1287 DAPI positive cells were analyzed for VLR-Fc-RBC36 and -30 respectively, *p* < 0.001 = ***determined using one-way ANOVA.

Although the aforementioned data demonstrate that each of the 3 VLR-Fcs are capable of trafficking across the MBECs, the unequivocal ultrastructural detection of parenchymal VLR-Fc upon full transcytosis into brain is challenging. An antibody undergoes substantial dilution upon exiting the vesicular trafficking network of MBECs and entering the brain parenchyma, rendering it largely undetectable by ultrastructural imaging^47^. However, the presence of a secondary cell target can serve as a site where a transported antibody is reconcentrated and can be readily detected after penetration into the parenchyma (e.g. anti-TfR binding to postvascular cells^11,21,48^). VLR-Fc-30 also binds to parenchymal resident cells, including neurons, in addition to trafficking across the MBEC (Fig. S4), and this secondary cellular targeting facilitates evaluation of full VLR transcytosis. Postvascular cell targeting of VLR-Fc-30 was quantified 11 h following IV administration and compared to negative control VLR-Fc-RBC36. Two main postvascular labeling patterns were identified. First, there were fairly rare instances of distinct lectin-negative cells within the brain parenchyma that were strongly labeled with VLR-Fc-30 (~ 1% cells, fully positive cells, Fig. 9A,B), whereas these events were not observed for VLR-Fc-RBC36. Second, there were parenchymal cells with punctate VLR-Fc-30 labeling that was often perinuclear (~ 12% cells, Fig. 9A,B). Combined, these data suggest that VLR-Fc-30 is able to fully transcytose the BBB and enter the brain parenchyma.

## Discussion

In this work, we have deployed a unique combination of lamprey immunization, yeast display screening and antibody binding characterization techniques to identify a panel of lamprey VLRs that are capable of binding antigens present at the brain microvasculature in vivo, with a subset of these being capable of homing to and trafficking at the BBB after systemic administration. The novel multi-tiered screening strategy developed and applied in this study combined the creation of an immunized library with elements of previously reported YSD screening approaches^18,49–53^ in order to rapidly isolate VLRs that target MBECs in vivo. Indeed, since lamprey were immunized with PM fractions prepared from freshly isolated mouse brain microvessels, a robust polyclonal immune response against in vivo-relevant BBB antigens was generated, similar to that reported with other antigen sources^54^. Porting the immune lamprey VLR repertoire into the YSD platform enabled clonal screening of the immune response to BBB PM antigens. Previous work carried out in our lab has established the YDIP procedure as a platform for discovery and optimization of antibodies against membrane protein targets through screening of combinatorial YSD libraries using detergent solubilized lysates of cultured cells as sources for antigen binding and competition steps^40,50,51^. Similarly, others have had success using VLRS in YSD format^55^. In this work, we extended the platform by employing detergent-solubilized antigen preparations derived directly from freshly isolated mouse brain microvessels, thereby increasing the in vivo relevance of the antigens presented during VLR library screening. It is well known that expression profiles are altered when BBB cells are cultured out of their natural environment^19,20^; therefore, screening with antigens derived from in vitro cultured cells alone often yields a large proportion of antibodies that lack in vivo relevance^18,56^. Consequently, two rounds of YDIP enrichment with in vivo antigen preparations were coupled to downstream biopanning screening to filter the VLRs and identify those that could bind cell surface antigens. YSD biopanning on live MBEC monolayers eliminated the possibility of VLRs interacting with intracellular epitopes of integral membrane proteins or membrane-associated intracellular machinery that were part of the PM preparations used for immunization and YDIP, strongly biasing enrichment towards VLRs targeting cell surface-exposed epitopes. The coupling of YDIP with biopanning was quite successful as non-exhaustive sampling of the biopanning output pool (roughly 200 VLR displaying yeast clones were tested) indicated that around 85% specifically recognized MBEC cell surface antigens, representing 33 unique VLR sequences. Importantly, ~ 60% (16 out of 26 that could be tested) of those VLRs testing positive in in vitro binding assays also bound their target in mouse brain microvessels (Table S1). This represents a significant improvement over a previous study from our laboratory in which less than 5% of in vitro binding antibodies recognized in vivo antigens when biopanning alone was used to enrich a library for BBB binders^18^. Finally, while we used immortalized MBECs as a screening substrate to identify extracellular targets (Fig. 2, step iv), it may be possible to identify additional VLRs using primary MBECs since it is possible that the primary cells may express targets that have been downregulated in the immortalized cells.

Next, brain microvasculature-binding VLRs were tested for their capacity to target receptor-mediated endocytosis machinery, an important attribute for brain drug delivery applications. Of 16 VLRs shown to bind brain microvessels in tissue sections, 4 of these clones had endocytosis capability. This frequency of cell binders capable of receptor-mediated internalization compares favorably with previous yeast biopanning experiments where approximately 25% of unique cell-surface binders were found to endocytose^18^. Importantly, in vitro internalization behavior was predictive of internalization in vivo as endocytosis and trafficking of IV administered VLR-Fc-11, -30, and -46 was confirmed through confocal and electron microscopy analyses. Although the antigens recognized by the 16 unique microvessel binding VLRs have not yet been fully investigated, differential cell surface binding, intracellular localization and brain section staining patterns, as well as sequence diversity of the VLR clones, suggests that they recognize a varied set of BBB antigens.

One of the main motivations for employing VLRs is that VLRs have proven robust in binding various glycan antigens with desirable affinity and specificity^29,30,32,45,54^. This is especially relevant to BBB-targeting applications as a multitude of known BBB transporters, such as glucose transporter, GLUT1^37^, are glycoproteins, and the BBB glycocalyx has been shown to play important roles at the BBB in health and disease. The BBB glycocalyx is a complex ~ 100 nm thick structure^57^, that has been suggested to serve roles in sieving molecules based on charge and size and therefore contribute to the selective permeability of the BBB^36^. For example, disruption of the BBB glycocalyx by heparinase treatment has been shown to increase BBB permeability and perfusion^37,38^. Moreover, the glycocalyx can play a mechanosensing role that can drive BBB responses to shear stress^58^. Accordingly, heterogeneity in adhesion molecule glycosylation has been suggested as a vascular and disease-specific molecular zip code for inflammatory responses^59^. It has also been suggested that the glycocalyx may play an important role in transcytosis at the BBB^60^. Despite the established importance of the BBB glycocalyx, the discovery and development of BBB-targeting reagents recognizing BBB glyco-epitopes has been under-pursued, perhaps because of the suboptimal glyco-recognition of Ig-based binding scaffolds that have been used in BBB screens^18,61^. However, there exists some evidence that targeting of glycostructures may be an effective epitope space for BBB targeting and delivery. For instance, the FC5 camelid antibody that is currently under development for brain delivery applications^62^ was found to be partially dependent on glycan binding^15^. In addition, there is an increasing literature describing brain glycomics, and many species, sex, age, or disease differences are likely to be discovered ^63,64^. Here, we focused on murine screening and subsequently confirmed human cross reactivity, but species and disease specific glycan profiles could be identified using a similar workflow by changing the lamprey immunogen or the screening subtrates^63^. In the current study, not only was the polyclonal lamprey serum shown to bind a whole host of glycans, the three lead VLRs, VLR-Fc-11, VLR-Fc-30 and VLR-Fc-46, all displayed a demonstrable glyco-signature. VLR-Fc-11 and VLR-Fc-46 recognize a Neu5Acα2-6Galβ1-4GlcNAcβ1-3Gal motif (as seen in Glycan ID 595, 329, and 327) whereas VLR-Fc-30 most strongly recognized a Neu5Acα2-6Galβ1-4GlcNAcβ1-3GalNAc motif (as seen in Glycan ID 373). The sialidase data suggest that some binding is retained even after sialic acid removal, suggesting a proteinaceous contribution to the antigenic epitope. It is possible that this glyco-signature is what is able to imbue these VLRs with their relatively specific brain vascular targeting ability when compared with other organs (Figs. 7B,C, S9). Given the novelty of using glycan-binding proteins for BBB targeting, these possibilities warrant further investigation. Interestingly, the glycan motifs recognized by the lead VLRs were not those that were strongly recognized by the polyclonal antiserum, indicating that low abundance VLRs can be enriched and isolated from the polyclonal repertoire using the methods described here. While outside of the scope of this work, the identification of the proteinaceous contribution to VLR-Fc binding^65,66^ is of great interest. Commonly used pull down methods to identify receptor-ligand interactions are notoriously complicated for membrane tethered proteins^67,68^. However, expression cloning methods^25^ and rapidly advancing gene editing approaches could be deployed to determine the specific targets recognized by the VLR-Fcs^69^.

The unique glyco-recognition attributes and the ability of VLR-Fc-11, -30 and -46 to engage and traffic within mouse BBB endothelium after IV administration indicate that the lead VLRs identified here have potential as novel constructs for applications in targeting therapeutics to the BBB. Given its secondary cellular targeting ability, VLR-Fc-30 was also demonstrated to fully transcytose the BBB and accumulate in postvascular cells, including neurons. Although VLR-Fc-11 and -46 do not have the secondary cellular target and thus could not be detected in postvascular tissue using ultrastructural imaging methods, their ability to traffic into and across BMECs in vivo was clear using immunocytochemistry and electron microscopy. Moreover, all three VLR demonstrated cross-reactivity to both mouse and human antigens, and immunhistochemical biodistribution results suggest the relative specificity of these VLRs for the brain vasculature.

Of course, future work to characterize pharmacokinetics, cellular trafficking, biodistribution, immunogenicity, and the ability to deliver pharmacologically relevant concentrations of drug payloads to the brain will be necessary to fully assess the translational promise of these novel VLRs. The engineering of antibody affinity and valency has been demonstrated to enhance brain penetration^8–10,12,65,70^, by changing subcellular trafficking to aid in transport across the endothelium^71,72^. VLR and VLR-based scaffolds have proven amenable to protein engineering techniques aimed at altering binding properties^54,66^, so these avenues are available if optimization of brain targeting and trafficking is required. Although VLRs remain underexplored for therapeutic applications, recent studies have demonstrated that Repebodies^73^, consensus designed LRR domain proteins based on VLRs, can mediate therapeutic outcomes in animal models motivating further exploration of VLRs as novel alternatives to traditional Ig-based therapeutics^66,74^. Thus, VLR-Fc-11, -30, and -46 are promising lead candidates that may offer unique alternatives for brain drug delivery applications.

## Materials and methods

### Cells, media, and plasmids

*Saccharomyces cerevisiae* strain EBY100 was used for VLR surface display. The plasmid used for VLR library cloning and display was pCT-ESO^75^. VLR RBC36^31^ which specifically recognizes the human blood group type II H trisaccharide (Fucα1,2-Galβ1,4-GlcNAc) was used as an isotype control where indicated. For all yeast surface display experiments, EBY100 yeast were first grown overnight at 30 °C 260 rpm in SD-CAA media (20 g/L dextrose, 6.7 g/L yeast nitrogen base, 100 mM sodium phosphate buffer pH 6.0, 5.0 g/L bacto-casamino acids without tryptophan and uracil). The day before an experiment all yeast cultures were re-set to an OD_600_ of ~ 0.4 and grown for 3–4 h until reaching an OD_600_ of 1. Then, surface display was induced via switching to SG-CAA induction media (same recipe as SD-CAA except galactose is used instead of dextrose) and cultures were grown at 20 °C, 260 rpm for 16–18 h. HEK293F cells were purchased from ATCC (CRL-1573) and maintained in Freestyle F17 Medium (Thermo Fisher) at 37 °C, 8% CO_2_, and 135 rpm in a humidified incubator. The plasmid used for production of soluble VLR-Fc was pIRES-VLR-Fc. bEnd.3 cells at passage 22 were purchased from ATCC (CRL-2299) and maintained in complete growth media (DMEM supplemented with 4 mM l-glutamine, 4500 mg/L glucose, 1 mM sodium pyruvate, 1500 mg/L sodium bicarbonate, and10% fetal bovine serum) at 37 °C, and 5%CO_2_ in a humidified incubator up to passage 30.

### Animals

Male C57BL/6 mice (Mus musculus) at 6 to 7 weeks of age were purchased from Envigo and used in terminal experiments. All mouse experiments were approved by the UW-Madison Institutional Animal Care and Use Committee (IACUC) and performed in compliance with the UW-Madison IACUC and following National Institutes of Health (NIH) guidelines for care and use of laboratory animals. Sea lamprey larvae (Petromyzon marinus) captured from the wild by commercial fishermen (Lamprey Services, Ludington, MI) were maintained in sand-lined, aerated aquariums at 16–20 °C and fed brewer’s yeast. All lamprey experiments were approved by the Emory University IACUC and performed in compliance with the Emory University IACUC and following National Institutes of Health (NIH) guidelines for care and use of laboratory animals. All experiments and data analysis were performed in accordance with the ARRIVE guidelines.

### Human tissue

De-identified normal human brain tissue was obtained from surgeries for other indications. All research was carried out in compliance and under the supervision of the University of Wisconsin-Madison Institutional Review Board and following the guidelines of the federal Common Rule. Patients give informed consent for surgery at the University of Wisconsin Hospital including a consent provision for the research use of leftover tissue removed during surgery.

### Capillary isolation, plasma membrane fractionation, and quality analysis

Brains were removed from 6–7 week old male C57BL/6 mice (~ 20 g) and stored in DMEM on ice. Microvessels were isolated and endothelial plasma membranes fractionated essentially as previously described^40^. Briefly, the cerebellum and white matter were dissected away and brains were rolled on Whatman 3MM chromatography blotting paper to remove the meninges. Up to 15 brains were homogenized in 20 mL DMEM + 0.2%BSA in a dounce homogenizer, and the homogenate was passed over a 150 μm nylon mesh to remove large debris. The homogenate was mixed with an equal volume of 40% dextran solution and centrifuged at 5000×*g* for 15 min at 4 °C. The supernatant was discarded, and the crude microvessel pellet was resuspended in DMEM + 0.2% BSA. Microvessels were then recovered on 41 μm nylon mesh filters and washed twice with PBS. To prepare biotinylated plasma membrane proteins for yeast display library screening, microvessel membrane proteins were biotinylated prior to plasma membrane fractionation via incubation with 5 mM sulfo-NHS-LC-biotin (Thermo Fisher), which is membrane impermeable, for up to 2 h at 4 °C. Unreacted biotinylation reagent was quenched by addition of glycine to a final concentration of 100 mM and 10 min incubation on ice. Biotinylated microvessels were washed twice with PBS + 100 mM glycine to ensure complete quenching and removal of unreacted biotinylation reagent. Plasma membranes prepared for lamprey immunization were not biotinylated. Endothelial plasma membranes were fractionated from the purified microvessels via a two-step hypotonic lysis: (1) distilled water at 4 °C for 2 h and (2) 10 mM Tris–HCl pH 7.4 at 4 °C for 30 min. This was followed by sonication in 50 mM Tris–HCl pH 7.4 and centrifugation at 25,000×*g*. This resulted in a supernatant containing dispersed plasma membrane fragments and a pellet containing the capillary basement membranes. The supernatant fraction is referred to as brain capillary plasma membranes (BMPM) and used for lamprey immunization and yeast display screening. All buffers contained protease inhibitor cocktail (PIC, Roche, 11836170001) and 2 mM EDTA. Total protein concentration in all fractions was quantified using a BCA assay kit (Thermo Fisher) following the manufacturer’s instructions. This isolation procedure yielded 255 ± 35 μg of BMPM proteins from 15 mice. For quality analysis of the plasma membrane fractionation via western blotting, 10 µg of total protein from each fraction was separated via SDS-PAGE and transferred to nitrocellulose. Western blotting for brain capillary endothelial membrane marker Glut1 was carried out using a 1:1000 diluted rabbit anti-Glut1 (Thermo Fisher, PA1-46152). Western blotting for astrocyte endfoot marker GFAP (astrocyte endfeet are tightly associated with the basement membrane) was achieved with a 1:1000 dilution of mouse-anti-GFAP (BD Biosciences, 556329). Further quality analysis was achieved via γ-glutamyl-transpeptidase (GGT) activity assay as previously described^76^.

### Lamprey immunizations

Sea lamprey larvae were sedated with 0.1 g/L tricainemethanesulfonate (Tricaine-S; Western Chemical, Inc.), then injected into the coelomic cavity with 50 µg of BMPMs in 30 µl of PBS. Three lampreys were immunized a total of three times at two week intervals and blood was collected two weeks after the final immunization from lampreys euthanized with 1 g/L Tricaine-S. Approximately 200 µl of blood was collected in 200 µl of PBS containing 30 mM EDTA as an anticoagulant. Blood plasma and leukocytes were separated from erythrocytes by layering the blood on top of 55% Percoll and centrifugation at 400×*g* for 5 min. Erythrocytes pelleted to the bottom of the tube, while leukocytes collected at the 55% Percoll interface and plasma remained above the interface. Buffer was added to the plasma samples to a final concentration of 20 mM MOPS/0.025% sodium azide pH 7.5 and stored at 4 °C. Leukocytes were stored in RNAlater at − 80 °C until needed for VLRB cDNA library cloning.

### VLR library cloning

RNA isolated from total leukocytes using the Qiagen RNeasy kit was reverse transcribed into cDNA using SuperScript III reverse transcriptase (Invitrogen) and oligo-dT priming. VLRB transcripts were amplified from the leukocyte cDNA by nested PCR using KOD high-fidelity DNA polymerase (Novagen). The first round of PCR utilized primers to the 5′ and 3′ untranslated region, (5′-CTCCGCTACTCGGCCTGCA) and (5′-CCGCCATCCCCGACCTTTG), respectively. The second round of PCR used primers that amplified only the VLRB antigen-binding domain from the LRRNT (5′-GCATGTCCCTCGCAGTG) to the LRRCT (5′-CGTGGTCGTAGCAACGTAG), and 50 bp of sequence homology to the yeast surface display vector was added to each primer for cloning by in vivo homologous recombination in transfected yeast cells. PCR products were excised from 1% agarose gels, purified using the Promega Wizard gel extraction kit and eluted in water. The pCT-ESO-BDNF yeast surface expression plasmid was digested with NheI, BamHI and NcoI to linearize the vector and remove the BDNF insert. Prior to transformation with the VLR library, yeast were grown to log-phase in SD-CAA media 30 °C until the culture density reached ~ 1 OD_600_. The yeast cells were harvested by centrifugation at 1000x*g*, washed in Milli-Q water, then incubated in 10 mM Tris/10 mM DTT/100 mM LiOAC, pH 7.6 at 225 rpm 30 °C for 20 min. After the incubation, the yeast cells were washed in Milli-Q water and resuspended in 1 M sorbitol at 1 × 10^9^ cells/ml. 200 µl of yeast cells were mixed with 1 µg of digested vector and 2 µg of the purified VLRB PCR product and added to a 0.2 cm electroporation cuvette on ice. The yeast were electroporated at 2.5 kV (12.5 kV/cm) using a Biorad Micropulser. After electroporation, the yeast cells were incubated in a 1:1 mixture of 1 M sorbitol and YPD media (Fisher Scientific) at 30 °C for 1 h, then transferred to SD-CAA media. A small aliquot of the electroporated yeast cells was serially diluted in SD-CAA media and plated on SD-CAA agar plates to calculate the total number of transformants. Three electroporated samples were combined resulting in a library of 7.5 × 10^6^ VLR clones. Aliquots of the yeast library were stored at − 80 °C in 15% glycerol.

### YSD library screening with detergent solubilized BMPM proteins

VLR display libraries and control yeast displaying VLR-RBC36 were grown and induced as described above for each round of YSD screening. Two rounds of screening via the YDIP method were carried out as previously described^42^ with modifications. In each round, ~ 250 µg freshly isolated biotinylated BMPM proteins were solubilized in a final volume of 1 mL PBS containing protease inhibitor cocktail (Roche), 2 mM EDTA, 1 mM Biotin, 1% w/v BSA, and 1% v/v TritonX-100. To ensure complete solubilization of membrane proteins the mixture was incubated for 15 min at 4 °C and insoluble debris was removed via centrifugation. The first round of screening was carried out using a magnetic activated cell sorting (MACS) protocol^77^ to recover VLR binding to biotinylated BMPM antigens. Briefly, 2.1 × 10^8^ yeast, 30-fold excess of starting library size, were incubated with 1 mL detergent solubilized BMPMs for 2 h at 4 °C with rotation. Yeast were then washed twice with 1 mL ice cold PBS + 1% TX-100 + 1%BSA (PBSTXA) and once with ice cold PBS + 1% BSA (PBSA). Washed yeast were resuspended in 0.5 mL ice cold PBSA, then 50 µL streptavidin microbeads (Miltenyi, 130-048-102) were added, and the mixture was incubated at 4 °C with rotation for 30 min. Microbead-bound yeast were washed once with 1 mL PBSA and resuspended in 0.5 mL PBSA. The 0.5 mL microbead-yeast suspension was applied to an LS column (Miltenyi, 130-042-401) placed within a Midi-MACS separator magnet (Miltenyi, 130-042-302). The column was washed twice with 3 mL ice cold PBSA, removed from the magnet, and yeast were eluted via plunging with 3 mL SD-CAA media. Dilutions of the eluate were plated to count the number of yeast recovered and the remaining yeast regrown for subsequent screening. In the second round of screening fluorescent activated cell sorting (FACS) was employed to further enrich for BMPM binders. 5  × 10^7^ yeast were incubated with 0.5 mL detergent solubilized BMPMs for two hours at 4 °C with rotation. Full length VLR expression was detected via labeling with rabbit-anti-cmyc epitope (Thermo Fisher, PA1-981) followed by a goat-anti-Rabbit IgG-Alexa488 secondary (Thermo Fisher, A-11008). Binding to biotinylated BMPM antigens was detected by labeling with a mouse-anti-biotin (Labvison, BTN.4) followed by a goat-anti-mouse IgG-allophycocyanin (Thermo Fisher, A-865). 3  × 10^7^ labeled yeast were sorted on a Becton Dickson SORP FACSAriaII (University of Wisconsin Carbone Cancer Center) to recover yeast double positive for VLR expression and BMPM antigen binding, and the sorted yeast were expanded in SD-CAA (Fig. S10).

### YSD library biopanning

A two-step biopanning method was developed and applied to remove extracellular matrix (ECM) binding VLRs from the FACS-sorted library while enriching for VLRs that bind to extracellular epitopes using the bEnd.3 MBEC line. For each round, two substrates were used for biopanning. One 6-well plate containing decellularized ECM from bEnd.3 culture was prepared by growing cells to ~ 90% confluence then switching the cells to media supplemented with 5% ~ 500 kDa dextran sulfate (DxS, Acros Organics, 433240050) to promote robust ECM deposition^78^. After 4–6 days in D × S, cells were washed with PBS and plates were decellularized via a non-enzymatic protocol to leave behind intact ECM^79,80^. This plate was used in the ECM subtraction step. A second plate containing bEnd.3 cells grown to confluence under normal culture conditions was also prepared and used for the MBEC binding step. Prior to incubation with yeast both ECM and MBEC, plates were blocked for 30 min with PBSA at 4 °C. ECM subtraction was initiated by addition of induced yeast libraries or control yeast expressing VLR RBC36 suspended in PBSA into wells of the ECM plate at a density of ~ 0.85 × 10^6^ yeast/cm^2^. The plate was incubated with gentle rocking for 2 h at 4 °C. Non-binding yeast were recovered from the ECM subtracted plate after 2 washes with ice cold PBSA and immediately applied to the MBEC binding plate for 2 h at 4 °C with gentle rocking. Non-binding yeast were removed by 3 washes with ice cold PBSA, and MBEC binding yeast were then recovered by scraping the cells into SD-CAA media. Dilutions of the MBEC binding cells were plated to count the number of yeast recovered and the remainder were expanded for subsequent rounds of biopanning or individual clone analysis.

### VLR-Fc subcloning, production, and purification

VLR identified from the YSD screen were cloned into an expression vector, pIRES-VLR-Fc constructed from pIRESpuro2 (Clontech, 6937-1), by cloning rabbit IgG-Fc into the AgeI and BamHI sites. The expression vector included the VLRB signal peptide upstream of the multiple cloning site (MCS) to promote secretion of VLR into the culture media and the rabbit IgG-Fc downstream of the MCS to enable simple purification of VLR-Fc fusion proteins via ProteinA/G chromatography. VLR sequences were amplified via PCR with the following primers: VLRB-NT-NheI-F (5′-GAGAGCTAGCTGTCCCTCGCAGTGTTCG) and VLRB-CT-AgeI-R (5′-GAGAACCGGTCGTGGTCGTAGCAACGTAG). PCR products were digested with NheI and AgeI and ligated into the pIRES-VLR-Fc vector.

Soluble VLR-Fc fusion proteins were expressed by transient transfection of HEK293F suspension cultures. 80 µg pIRES-VLR-Fc plasmid DNA was mixed with 160 µg PEI (Polysciences, 23966) in 3 mL OptiPRO SFM (Thermo Fisher, 12309019) for 15 min and then applied dropwise to 80 mL HEK293F cultures. Transfected cultures were then incubated for 5–7 days at 37 °C, 8% CO_2_, 135 rpm in a humidified incubator and the supernatant containing secreted VLR-Fc was recovered via centrifugation and filtration. VLR-Fcs were purified from the cleared supernatant via gravity-driven chromatography over a packed bed of 100 µL Protein A/G Plus Agarose beads (Thermo Fisher, PI20423). After washing, three 200 µl fractions were eluted from the column with 100 mM Citric Acid pH 3 and neutralized with 1 M Tris-base pH 9, which typically yielded ~ 0.5 mg purified proteins from an 80 mL transfected culture. Purified proteins were stored for up to 2 months at 4 °C.

### Immunolabeling of tissue and cells with VLR-Fc

Fourteen micrometer coronal brain cryosections from male C57BL/6 mice were washed in PBS and then blocked and permeabilized with immunolabeling buffer (PBS + 10% goat serum + 1% BSA + 0.05% saponin) for 30 min at room temperature. Next, purified VLR-Fcs at 5 µg/mL in labeling buffer were incubated on the brain slices for 1–2 h at room temperature. After washing, brain sections were incubated with Goat-anti-Rabbit IgG-Alexa555 secondary to detect VLR-Fc binding and Isolectin B_4_-Alexa488 (Thermo Fisher, I21411) as a brain microvessel marker for 1 h on ice. After washing, sections were post-fixed with 4% PFA, nuclei labeled with DAPI, and mounted in ProLong Gold antifade reagent (Thermo Fisher, P10144). Cell surface binding on live bEnd.3 cells was carried out by incubation with 5 µg/mL purified VLR-Fc proteins in PBS + 10% Goat serum + 1%BSA (PBSGA) for 1 h at 4 °C. After washing VLR-Fc binding was detected by staining with Goat-anti-Rabbit IgG-Alexa555 in PBSGA for 30 min on ice. After washing, cells were post-fixed with 4% PFA, nuclei labeled with DAPI, and mounted in ProLong Gold antifade reagent. For whole cell labeling, cells were prefixed with 2% PFA, then blocked and permeabilized in immunolabeling buffer prior to incubation with VLR-Fc and detection reagents as described for cell surface binding. In all cases images were obtained with a Zeiss Imager Z2 Microscope equipped with an AxioCam MRm using 10 × or 63 × objectives. Human brain samples were obtained with approval from the University of Wisconsin-Madison Institutional Review Board, sectioned and labeled via the methods described above for mouse sections.

Co-labeling VLR-30-Fc and NeuN was done on 8 µm mouse brain cryosections labeled that had been transcardially perfused with DyLight488 conjugated tomato lectin (LEL, Vector Laboratories, DL-1174). Sections were labeled with VLR-Fc-30 as described above, with the exception that for this assay a VLR-human Fc fusion protein was used, and consequently the secondary antibody was a Goat-anti-Human IgG-Alexa 555. Following labeling and staining for VLR-Fc, sections were incubated with anti-NeuN antibody (Abcam, ab104225) overnight at 4 °C in PBSGA, the sections were then washed and labeled with Goat-anti-Rabbit IgG-Alexa 647 30 min in PBSGA before washing and mounting in ProLong Gold antifade reagent.

### Cell-based assays

*Internalization assays*– bEnd.3 cells analyzed by immunofluorescence microscopy were grown to confluence on glass coverslips. bEnd.3 cells used in quantitative internalization assays were grown to confluence in 96-well flat-bottomed plates (Corning, 353948). Cells were serum starved for 1 h at 37 °C in serum-free complete growth media. For endocytosis inhibitor experiments, 10 µg/mL (15.3 µM) fillipin, 20 µg/mL (62.7 µM) chlorpromazine, or 250 µg/mL (940 µM) amiloride were pre-incubated with the cells, transferrin-555 and cholera toxin subunit B-555 were used as controls. Subsequently, purified VLR-Fc diluted in serum free complete growth media were applied to the cells. Conditions were varied depending on the experiment. For temperature dependent internalization assays one group of cells was incubated with 10 µg/mL VLR-Fc at 37 °C and one group with the same concentration of VLR-Fc at 4 °C. Both groups were incubated for 30 min prior to subsequent labeling steps. For saturation experiments, all cells were incubated for 20 min at 37 °C with varying concentrations of VLR-Fc up to 4 µM. Samples for microscopy analysis were processed as follows. After the VLR-Fc incubation period, bEnd.3 cells were washed 3 × with ice cold PBS and incubated with Goat-anti-Rabbit IgG-Alexa488 in PBSGA for 30 min on ice to label cell surface bound VLR-Fc. Following washes, cells were fixed in 2% PFA for 10 min at room temperature and then blocked and permeabilized in immunolabeling buffer for 30 min on ice. To differentially label internalized VLR-Fc the fixed and permeabilized cells were incubated with Goat-anti-Rabbit IgG-Alexa555 in immunolabeling buffer for 30 min on ice. After washing, cells were post-fixed with 4% PFA, nuclei labeled with DAPI, and mounted in ProLong Gold Antifade Reagent. Samples were analyzed via widefield and/or confocal microscopy as described below. Quantification of the internalization ratio was done using ImageJ, the ratio of the total image intensity for the 555 internal VLR-Fc to 488 external VLR-Fc images was calculated and normalized to the control well for each VLR-Fc. Similarly, for the transferrin and cholera toxin controls, the total image intensity of the 555 channel was normalized to the control without inhibitor.

Samples for quantitative analysis of internalized VLR-Fc for temperature-dependent internalization measurements were processed as follows. bEnd.3 cells were first acid washed by 5 changes of ice-cold 0.9% w/v saline, pH 2.5 for a total of 25 min to remove cell-surface bound VLR-Fc. This stripping procedure routinely resulted in the removal of ~ 90% of the cell-surface bound VLR-Fc signal (Fig. S12). Cells were then fixed with 2% PFA and blocked and permeabilized in Odyssey Blocking Buffer (Li-Cor, 927-40000) + 0.1% TX-100 for 30 min at room temperature. Internalized VLR-Fc were detected by IRdye800CW Goat-anti-rabbit IgG (Li-Cor, 925-32211) and cell number in each well estimated with CellTag 700 (Li-Cor, 926-041090) both diluted in Odyssey Blocking Buffer and incubated with cells for 1 h at room temperature. After extensive washes with ice cold PBS + 0.1% Tween-20 and drying of the plate, signal in each well was measured with a Li-Cor Odyssey Imager with a focus offset of 3 mm and resolution of 169 µm. VLR-Fc signal in each well was normalized to a per cell basis via dividing by the CellTag 700 signal.

*Equilibrium Binding Measurements-*bEnd.3 cells were grown to confluence in 96-well flat-bottomed plates, washed 3X in PBS, and fixed with 2% PFA for 10 min at room temperature. Fixed cells were blocked and permeabilized as described above. Equilibrium affinity titration measurements were achieved via incubation of the cells with purified VLR-Fc diluted to a range of concentrations from 800 pM to 4 µM at room temperature for 2 h. After extensive washing with ice cold PBS + 0.1% Tween-20 cells were labeled for detection with the IRDye reagents and analyzed as described above. Fraction of cellular antigen sites bound by VLR-Fc was quantified using background subtracted per-cell binding signal and the data was fit to a bimolecular equilibrium binding model to determine the dissociation constant (K_D_).

*Competition assay*—recombinant receptor ecto-domain proteins (2 μM), rIR (R&D systems, 7544-MR), rLDLR (R&D systems, 2255-LD), and rTfR (Sino Biologics, 50741-M07H) were incubated with 200 nM VLR-Fc proteins in serum free complete growth media for 30 min and then applied to serum starved bEnd.3 cells in 96-well plates to allow for VLR-Fc binding to cell surface receptors. Plates were incubated at 4 °C for 2 h. After extensive washing with ice cold PBS cells were fixed with 2% PFA, permeabilized, labeled with IRDye reagents, and analyzed as described above.

*Sialidase pre-treatment assay-* bEND.3 cells were grown to confluence in 96-well flat-bottom plate. Cells were fixed in 4% PFA for 20 min at room temperature, washed in PBS, then blocked in Odyssey Blocking Buffer for 90 min at room temperature. In some cases, cells were pre-incubated with sialidase (P0720L, New England Biolabs) for 30 min at 37 °C to cleave glycans containing terminal sialic acid motifs. VLR-Fc (15 μg/mL) or biotinylated lectin (15 μg/mL) were added to the cells and incubated for 2 h at 4 °C to allow for binding. Lectins used were SNA (sambucus niagra agglutinin, Vector Labs, B-1305-2), MALII (Maackia Amurensis Lectin II, Vector Labs, B-1265-1), and ConA (Concanavalin A, Vector Labs, B-1005-5). After washing with ice cold PBS + 0.1% Tween-20, VLR-Fc were labeled with IRdye800CW Goat anti-Rabbit IgG (925-32211, Li-Cor), lectin with IRdye800CW Streptavidin (925-32230, Li-Cor), and cell number with CellTag 700 (926-041090, Li-Cor). The plate was washed with ice cold PBS + 0.1%Tween-20 and dried. Signal was detected with a Li-Cor Odyssey Imager with a focus offset of 3 mm and resolution of 169 μm. Binding signal in each well was normalized to cell number using the CellTag700 signal.

### In vivo VLR-Fc brain targeting experiments

Male C57BL/6 mice (~ 20 g) were injected intravenously with 10 mg/kg VLR-Fc or positive control anti-TfR (8D3, AbDSerotec) in PBS. 8D3 was used as a positive control to validate internalization of the VLR-Fcs. After 1 h of antibody circulation mice were deeply anesthetized and the thoracic cavity was opened and transcardial perfusion was initiated via insertion of a catheter into the left ventricle and clipping of the right atrium. Ice cold wash buffer containing Earle’s balanced salts, 20 mM HEPES, 1 g/L glucose, 10 g/L BSA, and 5 mg/L DyLight488 conjugated tomato lectin (LEL, Vector Laboratories, DL-1174) was perfused at 5 mL/min for 5 min with a peristaltic pump to wash away unbound antibodies and label the vessel lumen with lectin. Then perfusion fixation with room temperature 4% PFA was carried out at the same flowrate for 10 min. Upon completion of perfusion the brain, heart, liver, and kidneys were dissected, and flash frozen in liquid nitrogen or stored in ice cold PBS. For immunofluorescence analysis, brains were cryopreserved in OCT and stored at − 80 °C prior to sectioning. For electron microscopic analysis tissue was immediately cut into 150 μm thick coronal sections on a vibratome and stored in fixative containing 4% PFA and 0.01% glutaraldehyde overnight at 4 °C with gentle agitation.

### Sample preparation and immunofluorescence microscopy

Thirty or eight micrometer thick coronal brain sections were cut on a cryostat, and adhered to positively charged glass slides. Sections were washed with PBS to remove embedding compound and fixed with 2% PFA. Tissue was blocked and permeabilized in immunolabeling buffer for 30 min at room temperature. To visualize VLR-Fc in the brain sections, tissue was incubated overnight with goat-anti-rabbit IgG Alexa555 in immunolabeling buffer at 4 °C. In some cases, a goat-anti-collagen IV antibody (AB769, EMD Millipore) diluted in donkey immunolabeling buffer (goat serum replaced by donkey serum) was incubated on the sections for 2 h at 4 °C. Subsequently, sections were incubated with donkey-anti-rabbit IgG-Alexa555 conjugate and donkey-anti-goat IgG-Alexa647 conjugate in donkey immunolabeling buffer overnight at 4 °C. In all cases, sections were post-fixed in 4% PFA, nuclei labeled with DAPI, and mounted in ProLong Gold antifade reagent. Low magnification widefield images were obtained on a Zeiss Imager Z2 Microscope equipped with an AxioCam MRm using a 10 × objective, 100 × images were taken on an Olympus Ix70 microscope equipped with a Hamamatsu ORCA-flash4.0LT camera. Confocal imaging was performed on a NikonAR1 microscope using a Plan Apo λ 60 × oil objective with 1.4 numerical aperture and optical z-sections were obtained with a step size of 250 nm. Z-stacks were typically taken through a thickness of 5–10 μm. Images were 12-bit, 1024 × 1024 pixels, with a pixel size of 100, 110, or 120 nm. Maximum intensity projections of the Z-stacks were created using the Maximum Intensity Projection tool in NIS Elements (Nikon Metrology). In some cases, image contrast and brightness was adjusted for clarity of presentation using ImageJ. In these cases, all related images and controls were processed in an identical manner.

Quantification of vascular biodistribution was done using ImageJ. First, images were adjusted to threshold VLR-Fc-RBC36 negative control parenchymal binding and identical adjustment was subsequently performed for each VLR image. Regions of interest corresponding with positive lectin staining were outlined and the average fluorescence intensity corresponding to VLR-Fc vascular binding was measured. The background parenchymal intensity was also measured as an average of three small random regions within the parenchyma. A ratio of these values was used to determine relative vascular binding. The same quantification method was used for the naïve and IV injected tissues.

Quantification of postvascular accumulation of VLR-Fc-30 was done by counting the number of nuclei in each 8 μm brain tissue section field of view, assigning puncta to the nearest nuclei and counting the number of nuclei in each field of view with associated puncta, or counting those cells which showed a strong, whole cell labeling. This was performed on both VLR-Fc-30 and VLR-Fc-RBC36 injected animal tissue.

### Sample preparation and electron microscopy

Immunogold labeling of 150 μm vibratome sections from mice injected with VLR-Fc was carried out with reagents purchased from Electron Microscopy Sciences (EMS) essentially following the manufacturers protocols. After aldehyde quenching with 0.1% NaBH_4_, sections were permeabilized via incubation with 0.1% TritonX-100 in PBS for 30 min, and then blocked with AURION Goat Serum Blocking Solution (EMS, 25596) for 1–2 h at room temperature. Subsequently, sections were incubated with Goat-anti-rabbit IgG-Ultrasmall Gold conjugate (EMS, 25100) diluted in PBS + 0.2% AURION BSA-c (EMS, 25557) overnight at 4 °C with gentle agitation. Following extensive washing sections were fixed in 2% glutaraldehyde for 30 min. Silver enhancement was carried out using the R-Gent silver enhancement kit (EMS, 25520) following the manufacturer’s instructions to increase the size of the ultrasmall gold particles. Sections were post fixed in 0.5% Osmium Tetroxide, 1% potassium ferrocyanide in 0.1 M sodium phosphate buffer for 1 h at room temperature. After rinsing, sections were dehydrated through a graded ethanol series (35%, 50%, 70%, 80%, 90% for 5 min each, 95% for 10 min, and 100% for 30 min). The sections were then infiltrated via incubations with increasing concentrations of PolyBed812 in propylene oxide. After infiltration, sections were embedded in 100% PolyBed812 overnight at 60 °C in a drying oven. 100 nm ultrathin sections were cut using a Leica EM UC6 ultramicrotome and captured on Pioloform carbon-coated 1 × 2 Cu slot grids (EMS) and contrasted with Reynolds lead citrate and uranyl acetate. The sections were examined on a Phillips CM120 transmission electron microscope and images captured with a MegaView III digital camera (Olympus-SIS).

### Statistical analysis

Specific methods for statistical analysis have been indicated in the Fig. captions with the number of replicates analyzed in each condition. A combination of one-way ANOVA paired with Tukey’s post-hoc analysis and two-tailed students *t* test were used based on the experiment. Statistics were calculated using excel and GraphPad Prism.

## Supplementary Information

Supplementary Information.

## Data Availability

The data that support the conclusions in the paper are available within the paper or its associated supplementary content files.

## Abbreviations

BBB: Blood–brain barrier
BEC: Brain endothelial cell
BMPM: Brain microvessel plasma membrane
CNS: Central nervous system
CFG: Consortium for Functional Glycomics
ECM: Extracellular matrix
FACS: Fluorescence activated cell sorting
GGT: Gamma-glutamyl transpeptidase
GFAP: Glial fibrillary acidic protein
GLUT-1: Glucose transporter
IR: Insulin receptor
IV: Intravenous
rLDLR: Low-density lipoprotein receptor
MACS: Magnetic activated cell sorting
MBEC: Mouse brain endothelial cell
PM: Plasma membranes
TfR: Transferrin receptor
VLR: Variable lymphocyte receptor
YDIP: Yeast display immunoprecipitation
YSD: Yeast surface display
