# Supplementary material for: Identification of lamprey variable lymphocyte receptors that target the brain vasculature
Source: Sci Rep. 2022 Apr 11;12:6044. doi: 10.1038/s41598-022-09962-8 (PMC9001667; doi:10.1038/s41598-022-09962-8)
Supplement: Supplementary file 1 — Supplementary Information. [file 41598_2022_9962_MOESM1_ESM.pdf]

Supplementary Tables and Figures

Table S1: Summary of *in vitro* and *in vivo* characterization for select clones from the BBBVLR-BP3 pool

	Binds bEnd.3 cell surface in YSD format	Binds bEnd.3 cell surface in soluble format	Binds antigen in mouse brain capillaries	Binds antigen in mouse brain parenchyma	Temperature-dependent internalization in bend.3 cells	Binds lumenal antigen in vivo
**BBBVLR-BP3 Pool**	33/204	26/33	16/26	5/26	4/16	3/4
VLR-Fc-4	Yes	Yes	Yes	No	No	-
VLR-Fc-5	Yes	Yes	No	Yes	-	-
VLR-Fc-11	Yes	Yes	Yes	No	Yes	Yes
VLR-Fc-30	Yes	Yes	Yes	Yes	Yes	Yes
VLR-Fc-46	Yes	Yes	Yes	No	Yes	Yes
VLR-Fc-147	Yes	Yes	Yes	No	No	-
VLR-Fc-192	Yes	Yes	Yes	No	Yes	No

Table S2: Glycan binding measurements and glycan descriptions for the top 10 glycans recognized by VLR-Fcs-11, -30, and -46 in the CFG glycan microarray analysis.

	VLR-Fc 11		VLR-Fc-30		VLR-Fc-46			
Glycan ID	Average RFU	StDev	Average RFU	StDev	Average RFU	StDev	Glycan Description	Glycan Structure
**595**	8635	825	656	49	3335	726	Neu5Aca2-6Galb1-4GlcNAcb1-3Galb1-4GlcNAcb1-6(Neu5Aca2-6Galb1-4GlcNAcb1-3Galb1-4GlcNAcb1-3)GalNAca-Sp14	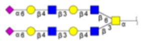
**329**	6635	771	922	61	4036	236	Neu5Aca2-6Galb1-4GlcNAcb1-3Galb1-4GlcNAcb1-3Galb1-4GlcNAcb-Sp0	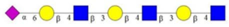
**327**	4486	451	805	41	874	72	Neu5Aca2-6Galb1-4GlcNAcb1-3Galb1-3GlcNAcb-Sp0	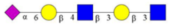
**596**	4036	333	885	40	2616	218	Neu5Aca2-6Galb1-4GlcNAcb1-3Galb1-4GlcNAcb1-3Galb1-4GlcNAcb1-2Mana1-6(Neu5Aca2-6Galb1-4GlcNAcb1-3Galb1-4GlcNAcb1-3Galb1-4GlcNAcb1-2Mana1-3)Manb1-4GlcNAcb1-4GlcNAcb-Sp12	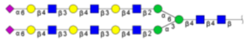
**587**	2663	323	367	31	1853	117	Neu5Aca2-6Galb1-4GlcNAcb1-3Galb1-4GlcNAcb1-3GalNAca-Sp14	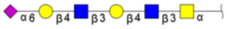
**269**	2624	318	295	11	883	58	Neu5Aca2-6Galb1-4GlcNAcb1-3Galb1-4GlcNAcb-Sp0	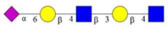
**591**	1300	36	380	89	534	55	Neu5Aca2-6Galb1-4GlcNAcb1-3Galb1-4GlcNAcb1-6(Galb1-3)GalNAca-Sp14	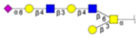
**345**	723	30	84	6	466	30	Neu5Aca2-6Galb1-4GlcNAcb1-2Mana1-3Manb1-4GlcNAcb1-4GlcNAc-Sp12	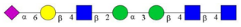
**57**	559	115	208	21	505	83	Neu5Aca2-6Galb1-4GlcNAcb1-2Mana1-6(Neu5Aca2-6Galb1-4GlcNAcb1-2Mana1-3)Manb1-4GlcNAcb1-4GlcNAcb-Sp24	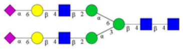
**477**	466	47	233	4	344	44	Neu5Aca2-6Galb1-4GlcNAcb1-2Mana1-6(Neu5Aca2-6Galb1-4GlcNAcb1-2Mana1-3)Manb1-4GlcNAcb1-4(Fuca1-6)GlcNAcb-Sp24	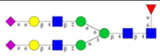
**323**	400	7	52	1	246	15	Neu5Aca2-3Galb1-4GlcNAcb1-2Mana1-6(Neu5Aca2-6Galb1-4GlcNAcb1-2Mana1-3)Manb1-4GlcNAcb1-4GlcNAcb-Sp12	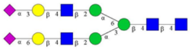
**373**	96	8	2597	342	25	2	Neu5Aca2-6Galb1-4GlcNAcb1-3GalNAc-Sp14	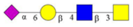
**546**	7	3	353	33	12	2	Neu5Gca2-8Neu5Gca2-6Galb1-4GlcNAc-Sp0	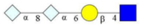
**513**	5	3	243	91	4	3	(6P)Galb1-4GlcNAcb-SP0	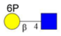

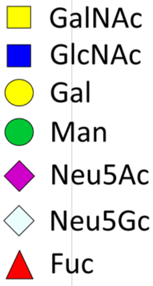

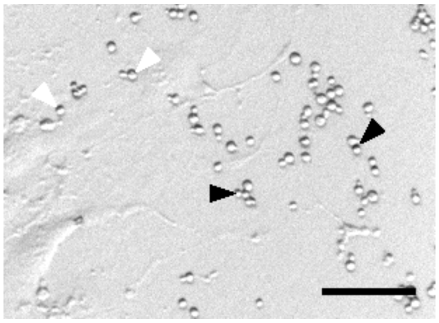

Figure S1: Biopanning experiment on sub-confluent MBECs reveals presence of a substantial population of ECM binding VLR in the library. Yeast (round phase dark cells) are found binding to both MBEC cells (white arrowheads), and ECM (black arrowheads). Scale bar = 50 μm.

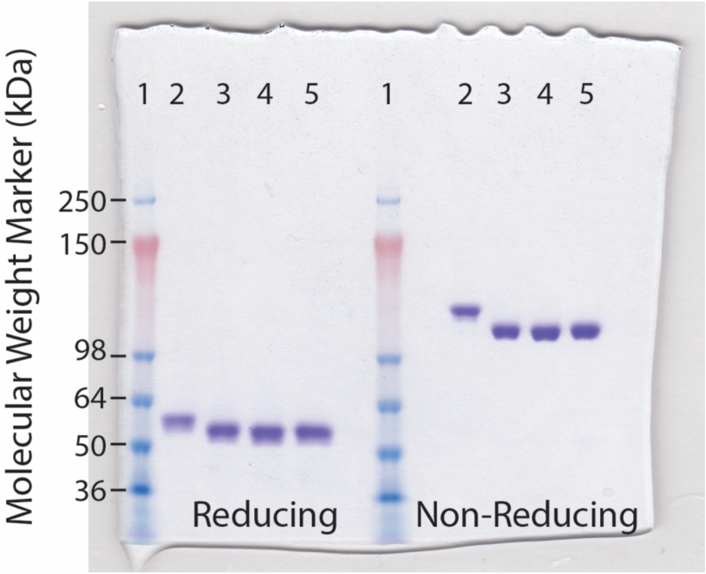

Figure S2: Coomassie-stained SDS-PAGE gel demonstrating that secreted and purified VLR-Fc having the expected monomeric (Reducing) and dimeric (Non-reducing) molecular weights. Lane 1: molecular weight marker, Lane 2: VLR-Fc-RBC36, Lane 3: VLR-Fc-11, Lane 4: VLR-Fc-30, Lane 5: VLR-Fc-46.

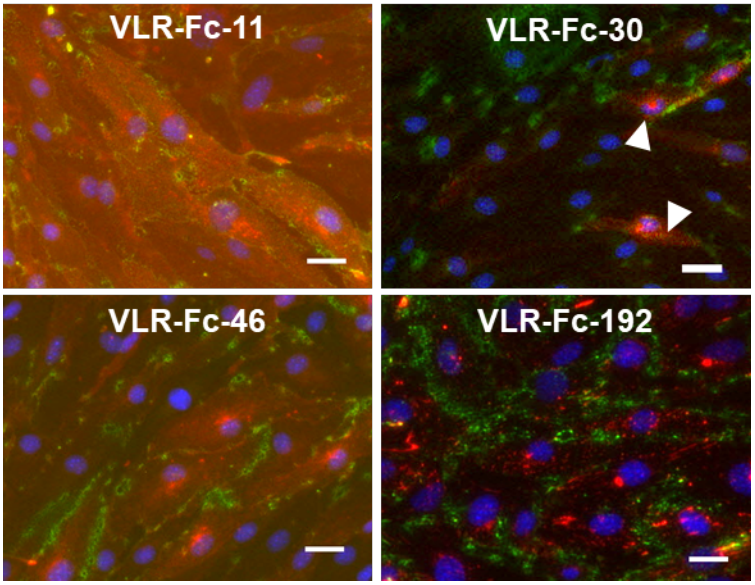

Figure S3: Fluorescence microscopy analysis of MBEC binding (green) and internalization (red). VLR-Fc-11, 46, and 192 are internalized by the majority of cells. On the other hand, only a small sub-population of MBECs are capable of internalization of VLR-Fc-30 (white arrowheads) while surface binding signal is seen throughout the plate. Red=internalized VLR-Fc, Green=Surface-bound VLR-Fc, Blue=DAPI. Scale bars=25 μm.

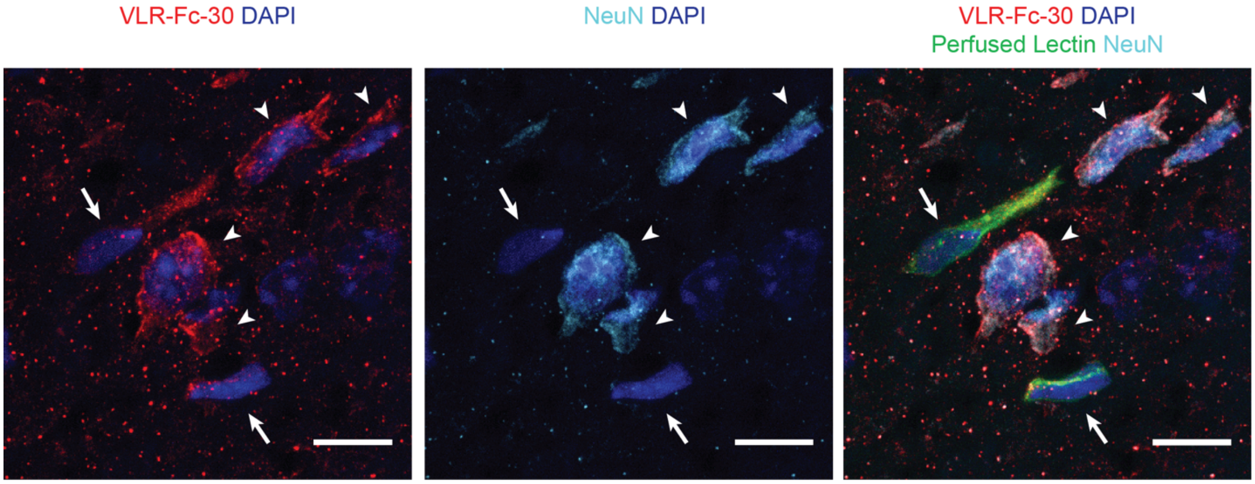

Figure S4: Mouse brain cryosections perfused with lectin (green) were labeled with VLR-Fc-30 (red) and anti NeuN (cyan) to show co-localization of VLR-Fc-30 and cells positive for NeuN, images are confocal maximum intensity projections. Vessels are indicated with arrows, neurons with arrow heads, scale bar is 10μm.

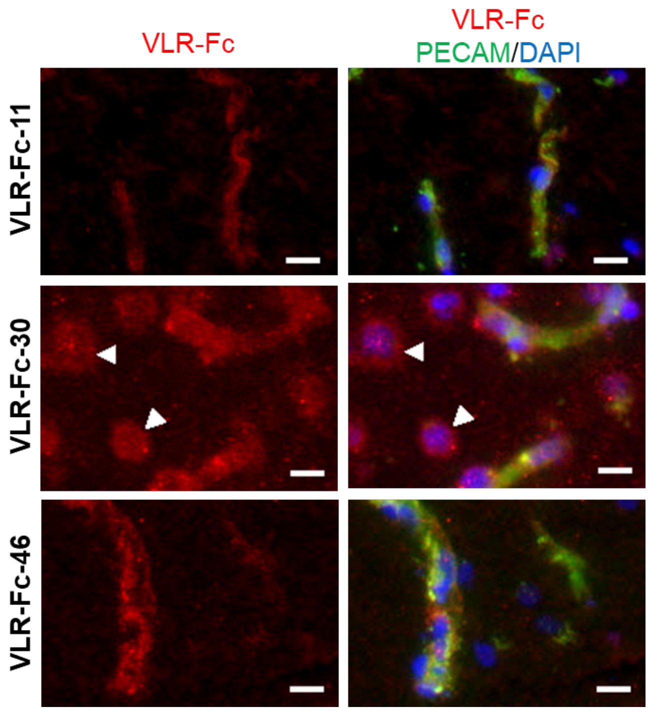

Figure S5: Immunofluorescence evaluation of VLR-Fc-11, -30, and -46 binding to human brain cryosections. VLR-Fc (red) and co-localization with PECAM-1 vascular label (green) and DAPI (blue) is shown. VLR-Fc-30 also recognizes antigens in brain parenchymal cells (white arrowheads). Scale bars=15 μm.

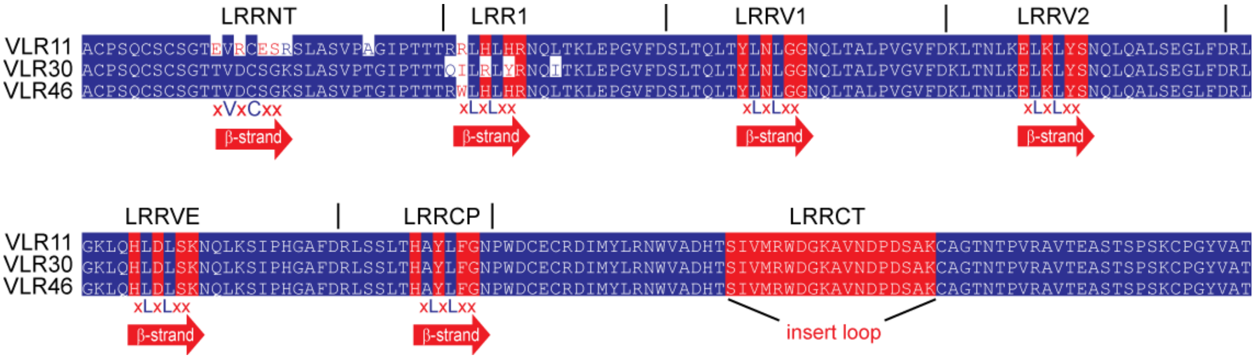

Figure S6: Annotated sequence alignment of VLR 11, 30, and 46. LRR domain designations are indicated above the sequences. Sequence regions corresponding to the β-strands that form the concave antigen-binding surface of the VLR are indicated below the sequences along with conserved amino acid positions in capital letters and variable positions indicated with a red x. Amino acid positions that are conserved between clones are indicated by blue or red highlight with sequence differences denoted by lack of highlight.

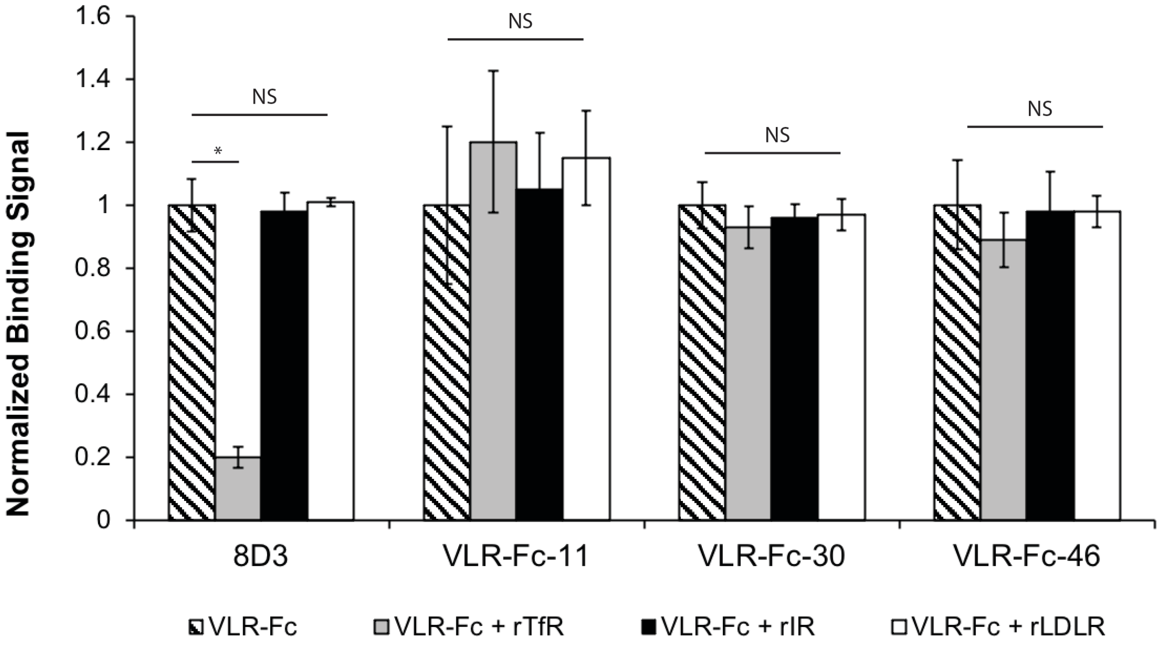

Figure S7: MBEC binding of VLR-Fcs with or without competition with soluble recombinant receptor ectodomains. VLR-Fcs are compared to the anti-TfR mAb 8D3. The only statistically significant difference is for 8D3 competition with rTFR. The mean±S.D. is plotted along with individual data points. A students t-test on n=3 replicates was used to determine statistical significance * = $p<0.05$, NS = not significant. rTfR = recombinant transferrin receptor, rIR = recombinant insulin receptor, rLDLR = recombinant low-density lipoprotein receptor.

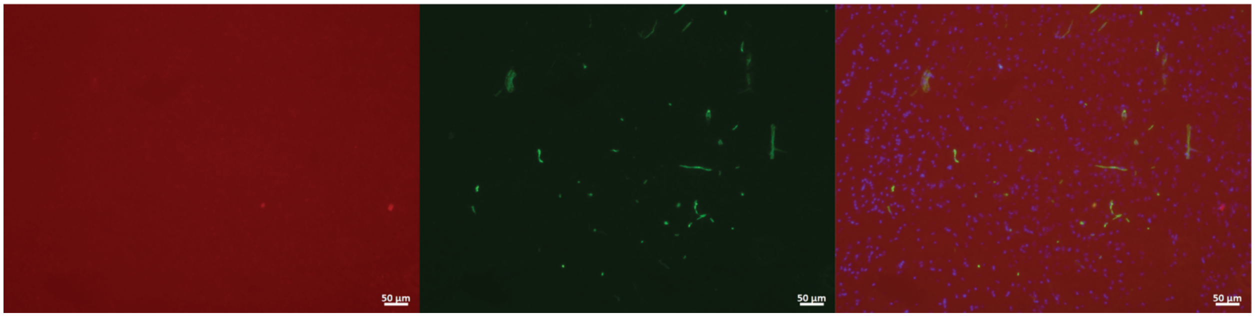

Figure S8: Mice were administered 10 mg/kg of VLR-Fc-192. After 1 hour of circulation time, mice were perfused with saline containing fluorescently labeled lectin to remove unbound VLR-Fc and label the vascular lumen. Low magnification of the cortex revealed no labeling with the injected VLR antibody construct.

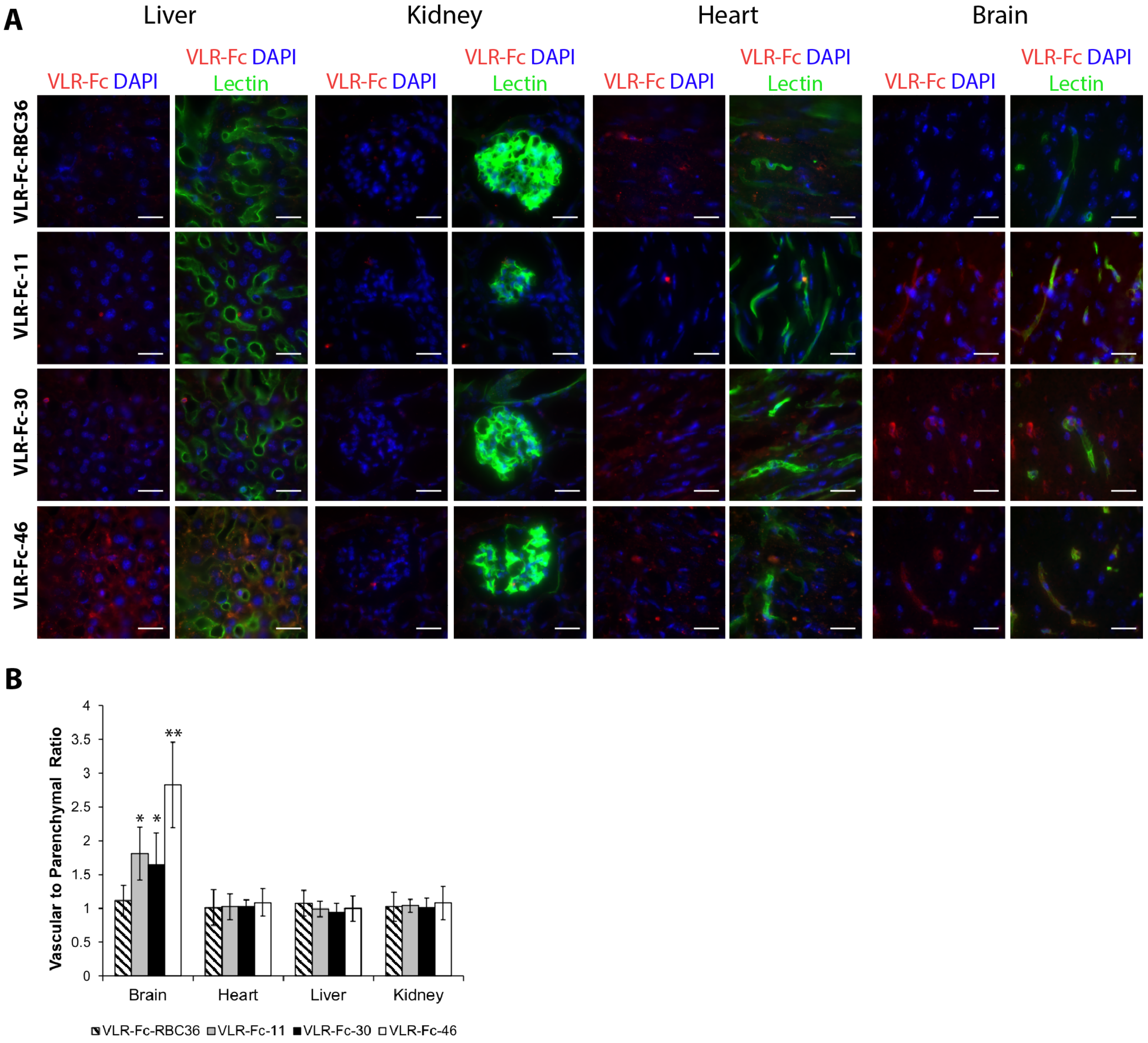

Figure S9: Immunofluorescence evaluation of vascular binding of VLR-Fc-11, -30, and -46 binding to naïve murine organ cryosections. (A) 5µg/mL VLR-Fc (red) incubated with naïve 8 µm cryosections of liver, kidney, heart, and brain and co-localization with perfused lectin vascular label (green) and DAPI (blue). Scale bars=25 µm. (B) Ratio of vascular to parenchymal VLR signal, mean ± standard deviation, $p < 0.01$ = **, $p < 0.05$ = * determined with one-way ANOVA paired with Tukey's post-hoc analysis, and images analyzed from at least 5 different regions of each organ.

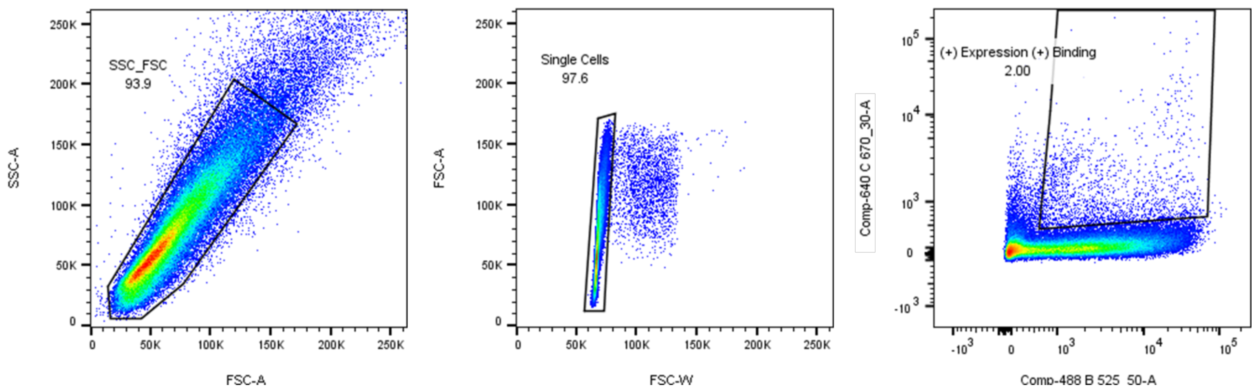

Figure S10: Gating strategy for FACS sorting of BMPM-binding VLR. After binding and labeling steps, yeast were analyzed and sorted on a Becton Dickson SORP FACSAriaII according to the gating strategy shown. Single cells were identified via SSC-A/FSC-A gating followed by FSC-A/FSC-W gating. From the single-cell population yeast clones that both expressed VLR (FL1, x-axis) and bound BMPM (FL4, y-axis) were isolated.

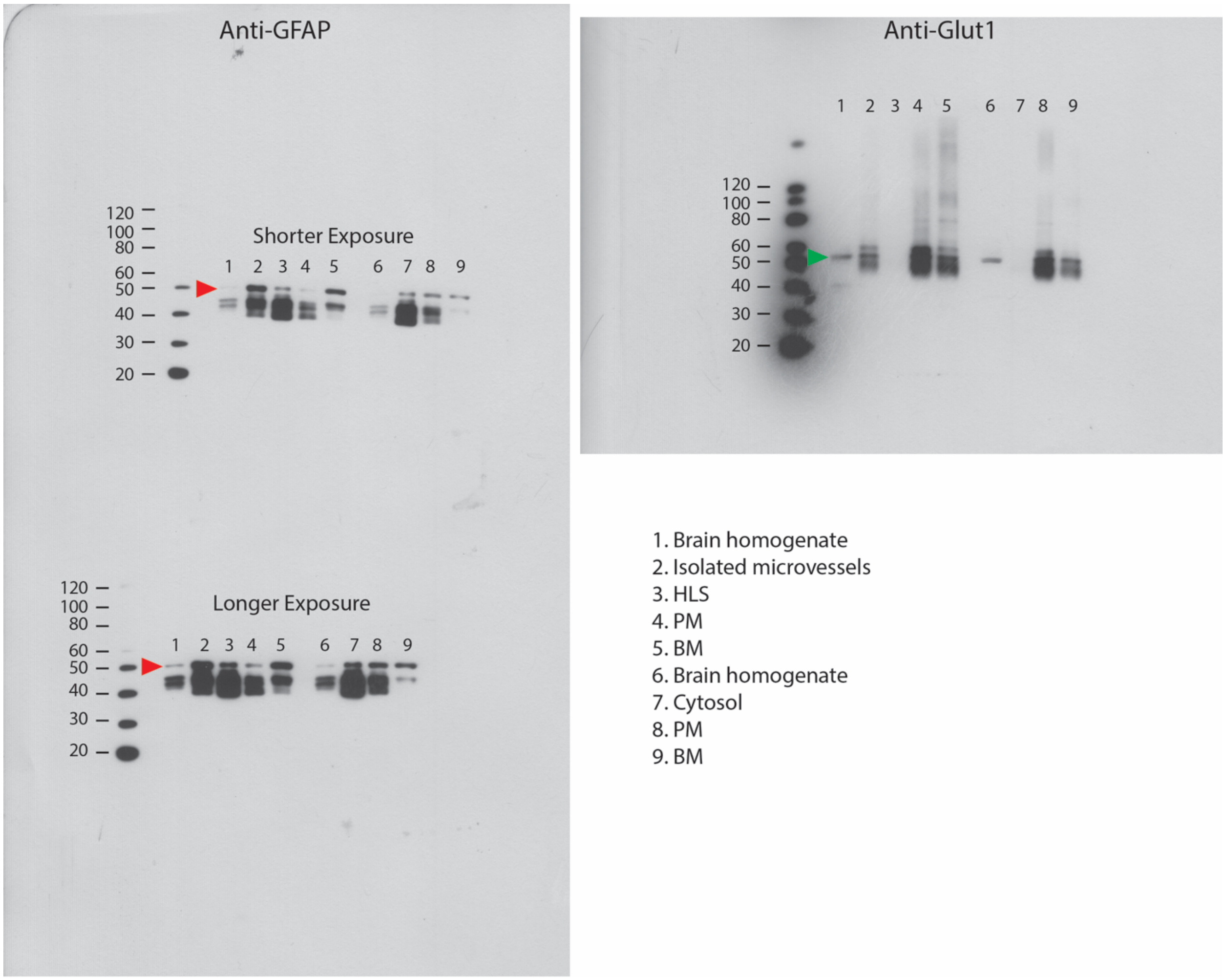

Figure S11: Western blot analysis of protein fractions from mouse brain microvessel isolation and plasma membrane fractionation for data used in Figure 1B. Green arrowhead highlights the major Glut1 band at ~55kDa while the red arrowheads highlight the major GFAP band at ~50kDa. Additional bands in Glut1 lanes likely correspond to differential glycosylation states. Additional bands in GFAP lanes likely correspond to breakdown products of the full-length protein. Molecular weights based on the MagicMarkXP Protein Standard are given in kDa on the left-hand side of each blot. The anti-Glut1 and longer exposure Anti-GFAP were used to create the cropped images shown in Figure 1. The shorter exposure anti-GFAP blot shows better visualization of the discrete full-length GFAP band (red arrowhead).

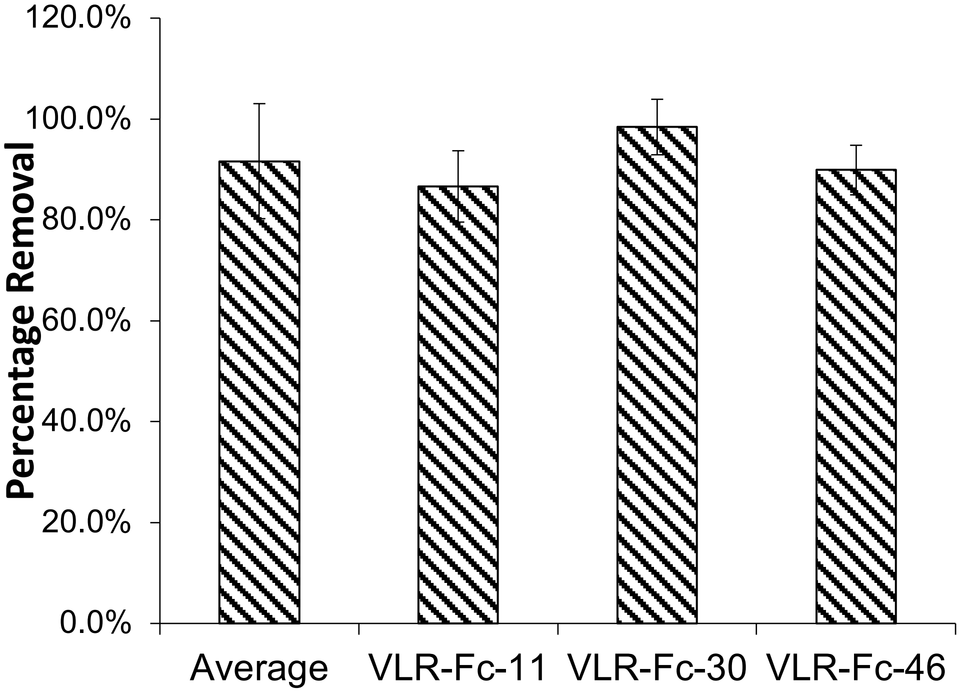

Figure S12: Efficiency of removal of surface bound VLR-Fc by stripping following internalization assay on bEnd.3 cells.
